# Supplementary material for: Predictive Minisci late stage functionalization with transfer learning
Source: Nat Commun. 2024 Jan 15;15:426. doi: 10.1038/s41467-023-42145-1 (PMC10789750; doi:10.1038/s41467-023-42145-1)
Supplement: Supplementary file 1 — Supplementary Information [file 41467_2023_42145_MOESM1_ESM.pdf]

Supporting Information

Predictive Minisci Late Stage Functionalization with Transfer Learning

Emma King-Smith, Felix A. Faber, Usa Reilly, Anton V. Sinistkiy, Qingyi Yang, Bo Liu, Dennis Hyek, and Alpha A. Lee*

Correspondence to: aal44@cam.ac.uk

Table of Contents

General Materials and Methods.. page S4
Table S1: Reagents Used for Each Reaction Type.. page S7
Table S2: Dataset Breakdown.. page S7
Calculating the Fukui Indices... page S8
Tie Breakers for F-scores.. page S9
Equations.. page S10
Figure S1.. page S11
Figure S2.. page S12
Figure S3.. page S13
Figure S4.. page S14
Figure S5.. page S15
Figure S6.. page S16
Compound Characterizations.. page S17-S26
Figure S7.. page S27
Figure S8.. page S28
Figure S9.. page S29
Figure S10.. page S30
Figure S11.. page S31
Figure S12.. page S32
Figure S13.. page S33
Figure S14.. page S34
Figure S15.. page S35
Figure S16.. page S36
Figure S17.. page S37
Figure S18.. page S38
Figure S19.. page S39
Figure S20.. page S40
Figure S21.. page S41
Figure S22.. page S42
Figure S23.. page S43
Figure S24.. page S44
Figure S25.. page S45
Figure S26.. page S46
Figure S27.. page S47
Figure S28.. page S48
Figure S29.. page S49
Figure S30.. page S50
Figure S31.. page S51
Figure S32.. page S52
Figure S33.. page S53
Figure S34.. page S54
Figure S35.. page S55
Figure S36.. page S56
Figure S37.. page S57

Supplementary References.. page S58

General Materials and Methods:[1]

Unless otherwise noted, all chemicals and reagents for chemical reactions were purchased at the highest commercial quality and used without further purification. Liver microsomes were purchased from the following vendors: female mouse, male rat, male cynomolgus monkey and non-transfected microsomes (Corning, Woburn, MA); dexamethasone- induced male rat, male hamster, male dog and pooled male & female human (prepared in-house at Pfizer, Groton, CT); and male guinea pig and male rabbit (Xenotech, Lenexa, KS). Note, human and monkey liver microsomes are considered biohazardous materials and appropriate precautions should be taken during handling and disposal. Recombinant human P450 enzymes heterologously expressed in microsomes from Sf9 cells were custom prepared by Panvera (Madison, WI).

Code:

The pipeline, modules, and trained models can be found at https://github.com/emmaking-smith/SET_LSF_CODE.

Liver Microsomes and Recombinant CYP Enzyme Screen Procedure:

Loratadine (20.0 μM, 10.0 nmol) was incubated with 9 mammalian liver microsomes (2.0 mg/mL) and 9 human recombinant P450 enzymes (2.0 mg/mL). The total volume in each well was 0.5 mL which contained potassium phosphate buffer (0.1 M, pH 7.4), MgCl2 (3.3 mM, 1.65 μmol) and acetonitrile (0.4% v/v). The reaction was initiated with the addition of NADPH (1.3 mM, 0.65 μmol) and agitated at 37 ˚C in a reciprocal shaking bath at 1" throw for 1 hour. The incubation was quenched with the addition of acetonitrile (1.5 mL), followed by centrifugation (1700 g, 5 min). The supernatant was removed and the solvent reduced using a Genevac centrifuge evaporator. The residue was reconstituted in 0.1 mL of 1% formic acid in H2O / 20% acetonitrile, followed by centrifugation (1700 g, 5 min). The samples were analyzed using the General HPLC-MS Method for Screen Analysis.

Biomimetic Metalloporphyrin Oxidation (BMO) Screen Procedure:

A high throughput BMO screen was completed on a miniature scale, examining the key variables of metalloporphyrin, oxidant and solvent. Nine different metalloporphyrins plus control without metalloporphyrin, six oxidants and two solvents were screened in a matrix of 120 combinations. The remaining variables were held constant according to the standard protocol described below.

The reactions were set-up in two 96-well arrays using miniature 8 x 20 mm (0.2 mL) glass vials under standard glove box conditions (H_2O and O_2 <20 ppm). A 8 x 20 mm (0.2 mL) glass vial equipped with stir bar was dispensed the reaction solvent (100 μL, 4.0 mM) followed by a solution of 1 (5.0 μL, 0.4 μmol), added as a 0.1 M solution in dichloroethane. Stirring was initiated before the metalloporphyrin (4.0 μL, 0.04 μmol) was charged, as a 10.0 mM solution in dichloroethane. The vial was treated with a 0.1 M solution of imidazole (2.4 mL, 0.24 μmol) in H_2O, followed by a 0.4 M solution of formic acid (4.0 μL, 0.16 μmol) in H_2O. Finally, the oxidant (8.0 μL, 0.08 μmol) was added as a 0.1 M solution in dichloroethane. The reaction vial was crimp sealed with a PTFE / Silicone / PTFE septa to the glove box environment before the

reaction was left to stir at 25 ˚C for 18 hours. After this time period, the reaction was diluted with acetonitrile (0.2 mL) and analyzed directly by UPLC/MS. The UPLC/MS method used a 0.1% AcOH / NH_4CO2H / H_2O gradient over 0.8 minutes, running from 5-95% acetonitrile using a Waters Acquity UPLC BEH C18 30 x 2.1 mm column at 100 ˚C with a flow rate of 2.5 mL/min and a detection wavelength of 210-360 nm. 0.5 µL injections were made directly from diluted reaction mixtures and ionization monitored in positive mode.

General Minisci Functionalization with Baran Diversinates™ Procedure:

To 1-dram pressure release vial containing Diversinate™ sulfinate reagent as sodium or zinc salts e.g., RSO_2Na or $(RSO_2)_2Zn$ (3 eq - 6eq), was added to a solution of the test substrate molecule (~2 µmol, 1 eq) in DMSO (~70-100 µL, 30 mM) and TFA (4 eq) followed by *tert*-butyl hydroperoxide, 70% in water (5 eq) at room temperature. The resulting reaction mixture was capped and heated to 50 ˚C overnight. The crude reaction mixture was dissolved in 3:1 acidic mobile phase (1% acetonitrile, 0.1% formic acid) and acetonitrile (~3 mL) then purified via HPLC (XSelect 5 µm C18 130 Å, 250 x 10 mm @ 2 mL/min). The respective fractions were pooled, and solvent removed using the EZ-2 Elite Genevac (3-hour HPLC setting, 34 ˚C / 238 mbar to 41 ˚C / 7 mbar). Each isolate was characterized by MS and NMR. Due to the low amounts of isolates generated, gravimetric mass analysis is not possible; qNMR in conjunction with the enhanced sensitivity using a 1.7 mm micro-cryoprobe in DMSO-d_6 solvent was used to determine the concentration of the sample.

General Minisci Functionalization with Molander BF_3K Salts Procedure:

To 1-dram pressure release vial containing the test substrate molecule (~2 µmol, 1 eq), potassium trifluoroborate salt of the radical (1.5 - 2 eq), in a 1:1 mixture of acetic acid and water to make a 30 mM solution and $Mn(OAc)_3$ was added in one portion. The resulting reaction mixture was capped and heated to 50 ˚C overnight. The crude reaction mixture was dissolved in 3 :1 acidic mobile phase (1% acetonitrile, 0.1% formic acid) and acetonitrile (~3 mL) then purified via HPLC (XSelect 5 µm C18 130 Å, 250 x 10 mm @ 2 mL/min). The respective fractions were pooled, and solvent removed using the EZ-2 Elite Genevac (3-hour HPLC setting, 34 ˚C / 238 mbar to 41 ˚C / 7 mbar). Each isolate was characterized by MS and NMR. Due to the low amounts of isolates generated, gravimetric mass analysis is not possible; qNMR in conjunction with the enhanced sensitivity using a 1.7 mm micro-cryoprobe in DMSO-d_6 solvent was used to determine the concentration of the sample.

Molecule Dynamics Simulations:

Molecule conformations were generated with MOPAC at the PM7 level of theory.[2] The underlying molecular dynamics (MD) driver was the Atomic Simulation Environment (ASE) package.[3] A Langevin thermostat controlled the temperature. First, the molecular geometry was optimized followed by equilibration to 500 K for 2.5 picoseconds with a timestep of 0.25 femtoseconds. Upon equilibration, conformations were sampled every 2 picoseconds from a production run of 200 picoseconds in the NVT ensemble at 500 Kelvin, using a timestep of 0.5 femtoseconds with the same thermostat. This yielded a total of 100 configurations per molecule.

The message passing neural network was based on MACE.[4] Interatomic distances were incorporated as edge features in the message passing neural network, however, angles were not part of the edge featurization and no universal node was used in this variation of the message passing neural network. A cutoff radius of 5Å was used.

Table S1: Reagents Used for Each Reaction Type

Reaction Type	*Reagent*	*Count*
Minisci*	$(CF_3SO_2)_2Zn$	233
	$(HCF_2SO_2)_2Zn$	137
	$cBuBF_3K$	50
	Selectfluor	39
	CF_3SO_2Na	28
	$CH_3CF_2SO_2Na$	22
	$HOCH_2SO_2Na$	20
	$MeOCH_2BF_3K$	18
	$(iPrSO_2)_2Zn$	16
	$iPrBF_3K$	15
	$(CF_3CH_2SO_2)_2Zn$	10
Photoredox*	NFSI	31
	O2	26
	tBu peracetate	18
Electrochem	H_2O	6
	CF_3SO_2Na	5
	$CH_3CF_2SO_2Na$	1

* = Reagents used more than 10 times shown

Table S2: Dataset Breakdown

	Minisci	*Zinc sulfinate Minisci*	*P450*	*Electrochem*	*Photoredox*
Number of Total Reactions	1928	463	642	12	93
Number of negative reactions	1056	155	74	0	34

Calculating the Fukui Indices:

Reactivity indices for electrophilicity and nucleophilicity for the *i*-th atom were computed by multiplying the corresponding Fukui index [F_i(+) or F_i(-), respectively] of the *i*-th atom by global electrophilicity / nucleophilicity for the given molecule.

Reactivity indices for radical reactions were taken to be equal to the corresponding Fukui indices F_i(0). Global nucleophilicity of the molecule was computed as 8 eV + HOMO, and its global electrophilicity as (HOMO+LUMO)2/(4(LUMO-HOMO)), where HOMO and LUMO are the HOMO and LUMO energies of the molecule.

Fukui indices of the *i*-th atom F_i(+), F_i(-) and F_i(0) were computed as differences between the atomic charge of the *i*-th atom in the original molecule q_i(N) with N electrons, the charge of the same atom after adding one electron to the molecule q_i(N+1), and the charge of the same atom after removing one electron from the molecule q_i(N-1):

$$F_i(+) = q_i(\mathrm{N}) - q_i(\mathrm{N}+1)$$
$$F_i(+) = q_i(\mathrm{N}-1) - q_i(\mathrm{N})$$
$$F_i(0) = \frac{q_i(\mathrm{N}-1) - q_i(\mathrm{N}+1)}{2}$$

For electrophilicity and radical indices, quantum chemical computations were run with PBE/6-311G, and for nucleophilicity with B3LYP/6-311G**. As partial atomic charges, Mulliken charges were used. These DFT functionals, basis sets and types of atomic charges were chosen by optimizing the predicting performance of the reactivity indices in S_NAr and EAS reactions of an internal dataset of small organic molecules (unpublished). Quantum chemical computations were run in Terachem.[5]

Tie Breakers for F-scores:

Whilst F-scores represented an excellent first pass, it did lead to degenerate best models for a variety of loss function weightings. We thus developed our own in-house metric, EKS metric, that was more discerning than the F-score. Like the F-score, this metric would provide a single number encompassing model accuracy and precision but would be more penalizing towards errors, elucidating model performance differences more easily (Figure S1 & S2). It would also address the challenge of accurate baselines when evaluating model performance. A model that predicts nothing reacts would receive and F-score of 0, and a model that predicts everything reacts would receive a non-negative score (Eq. S1). This is slightly unintuitive, as a model that predicts most atoms will not undergo functionalization is actually representing a more chemically correct understanding of reactivity. The most functionalized molecule only saw 30% number of sites reacting, or 70% of its atoms did not react. Indeed, most of the molecules in our dataset saw 1 reaction site of fewer.

Our in-house metric would give a low score if the model yielded a high number of false positives (FP) or false negatives (FN) and a high score if the model yielded a high number of true positives (TP) or true negatives (TN). The score should also be dependent upon the scarcity of predicted reactive sites. The value of each TP and TN should be dependent on the how frequently the model predicts positives and negatives, respectively. An overly cautious model which blindly guesses that all atoms are unreactive should receive a lower value for each TN it predicts compared to a model that is more judicious about its unreactive predictions. To this end, the following metric was used (Eq. S2). The variables, $pred_p$ and $true_p$ refer to the ratio of predicted positives to all reactive sites. A $pred_p$ of 1 indicates a model that predicts all sites react and a $pred_p$ of 0 indicates a model where every molecule is unreactive. As reference, on the retrospective test set, a perfect model would receive a score of 119, a model that predicts nothing reacts would receive a score of -1.83, and a model that predicts everything reacts would be given a score of -1455. If a prediction is only 3% incorrect, the model score drops to 72, and at only 5% incorrect the score becomes 46.5.

Equations:

Eq. S1: F-score = $\frac{2 \cdot \mathrm{TP}}{2 \cdot \mathrm{TP} + \mathrm{FP} + \mathrm{FN}}$

Eq. S2: score $= \mathrm{FP} \cdot \log(true_p) + \mathrm{FN} \cdot \log(1 - true_p) - \mathrm{TP} \cdot \log(pred_p) - \mathrm{TN} \cdot \log(1 - pred_p)$

Eq. S3: BCE Loss $= \sum_{i=0}^{n} w_i \cdot (y_i \cdot \log(x_i) + (1 - y_i) \cdot \log(1 - x_i))$

Eq. S4: BCE weight 1 $= x \cdot y \cdot \log(pred_p) + (1 - y) \cdot (1 - x) \cdot \log(1 - pred_p) + (1 - y) \cdot x \cdot \log(true_p) + y \cdot (1 - x) \cdot \log(1 - true_p)$

Eq. S5: BCE weight 2 $= [x \cdot y \cdot \log(pred_p) + (1 - y) \cdot (1 - x) \cdot \log(1 - pred_p) + (1 - y) \cdot x \cdot \log(true_p) + y \cdot (1 - x) \cdot \log(1 - true_p)] + [y \cdot x \cdot \log(true_p)) + (1 - y) \cdot (1 - x) \cdot \log(1 - true_p) + (1 - y) \cdot x \cdot \log(1 - pred_p) + y \cdot x \cdot \log(pred_p)]$

Eq. S6: BCE weight 3 $= 2[x \cdot y \cdot \log(pred_p) + (1 - y) \cdot (1 - x) \cdot \log(1 - pred_p) + (1 - y) \cdot x \cdot \log(true_p) + y \cdot (1 - x) \cdot \log(1 - true_p)] + [y \cdot x \cdot \log(true_p)) + (1 - y) \cdot (1 - x) \cdot \log(1 - true_p) + (1 - y) \cdot x \cdot \log(1 - pred_p) + y \cdot x \cdot \log(pred_p)]$

x = predicted values, *w* = weighting values, *y* = experimental values, always 0 or 1

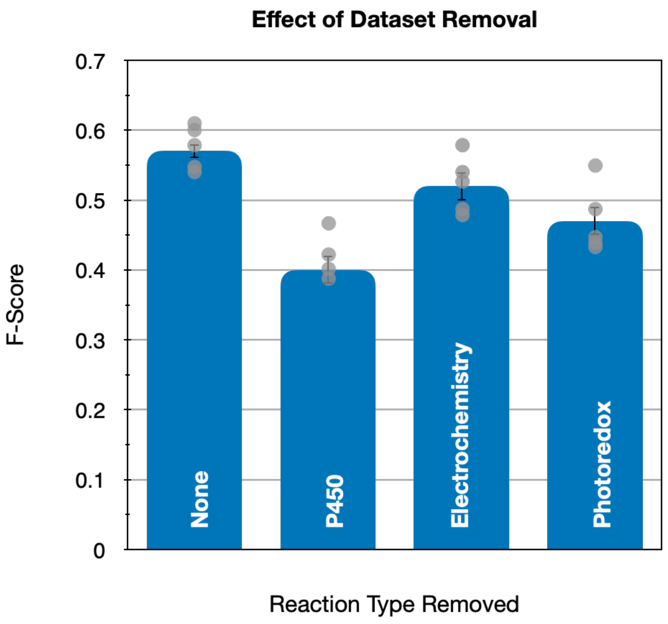

Figure S1: The deleterious effect of removing various reaction types from the training data (n=5). The bars in the bar charts represent the average with gray dots representing the individual data points (initializations with identical values are shown as a single point). Standard error bars shown. Source data for each bar chart can be found in source data excel file.

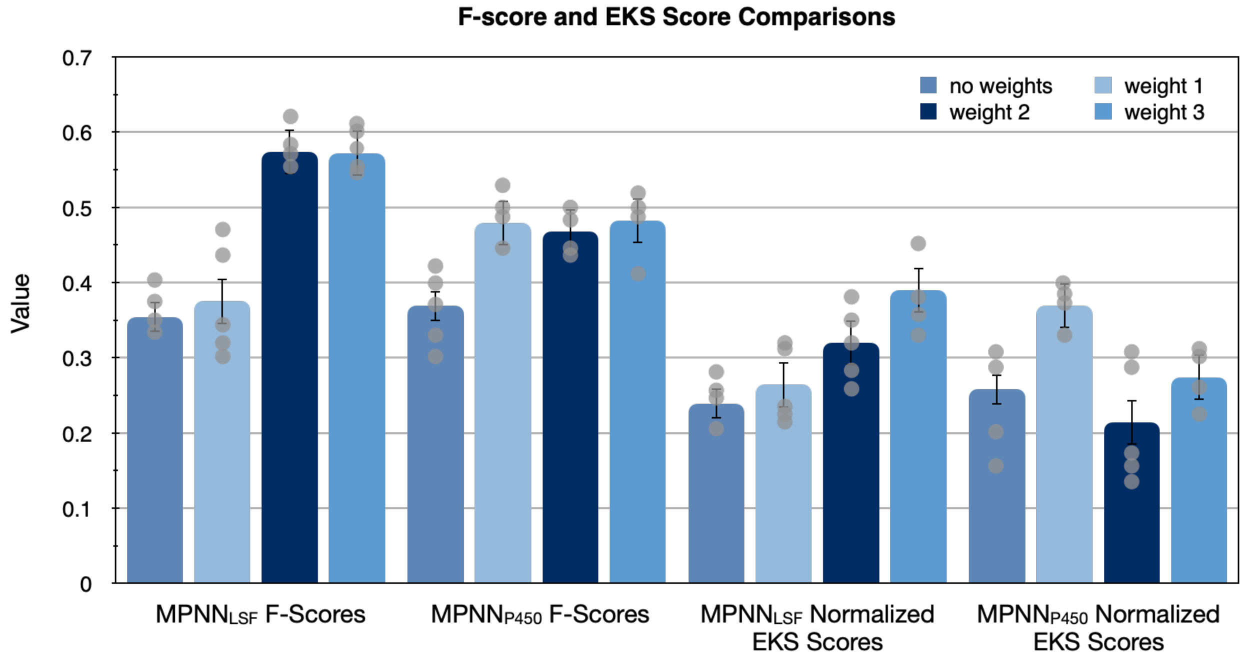

Figure S2: The comparisons of the best models', $MPNN_{LSF}$ (best model on retrospective test set) and $MPNN_{P450}$ (best model on P450-only test set) F-scores and normalized EKS scores at various Binary Cross Entropy Loss weightings on the retrospective and P450-only test set respectively (n=5). MPNN = message passing neural network. Notice that the normalized EKS scores show more discernment in model performance between different weightings. Weight 2 for $MPNN_{LSF}$ has a nearly identical F-score with weight 3, but EKS scores show a significant edge to weight 3. The bars in the bar charts represent the average with gray dots representing the individual data points (initializations with identical values are shown as a single point). Standard error bars shown. Source data for each bar chart can be found in source data excel file.

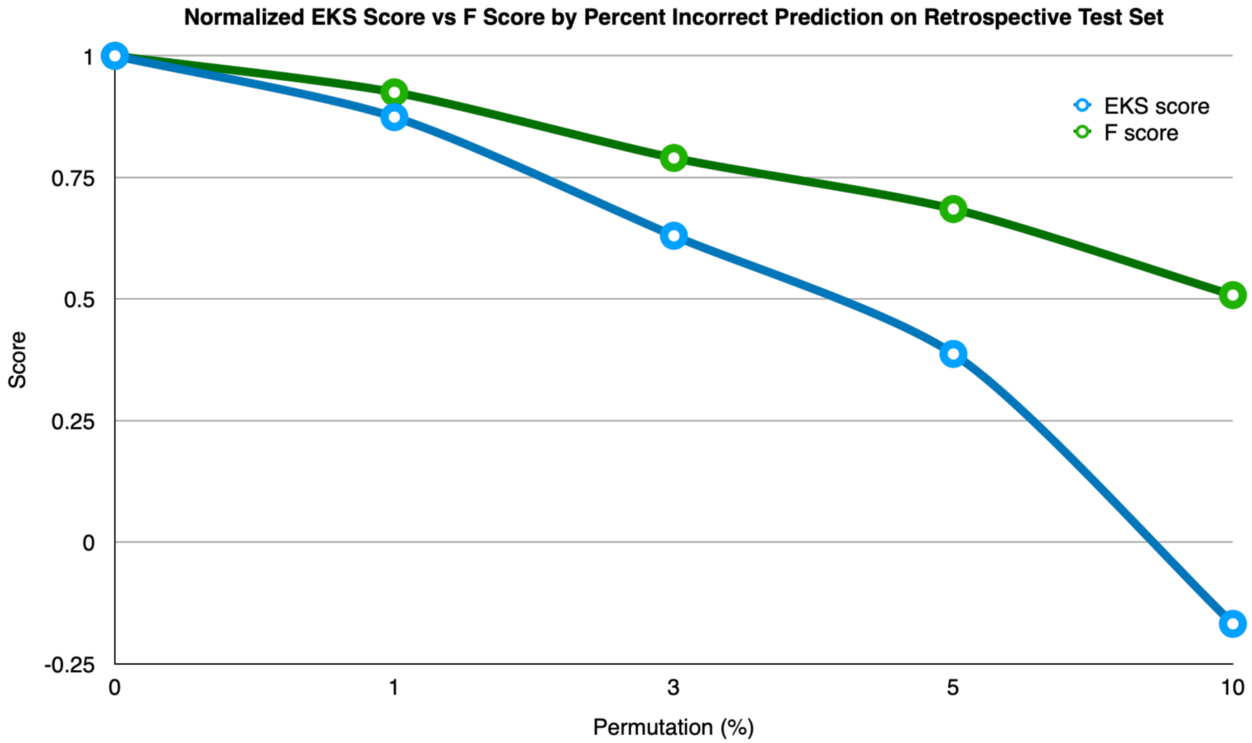

Figure S3: Comparison of sensitivity of incorrect predictions between EKS score and F-score. Analysis performed on the retrospective test set. Source data for each bar chart can be found in source data excel file.

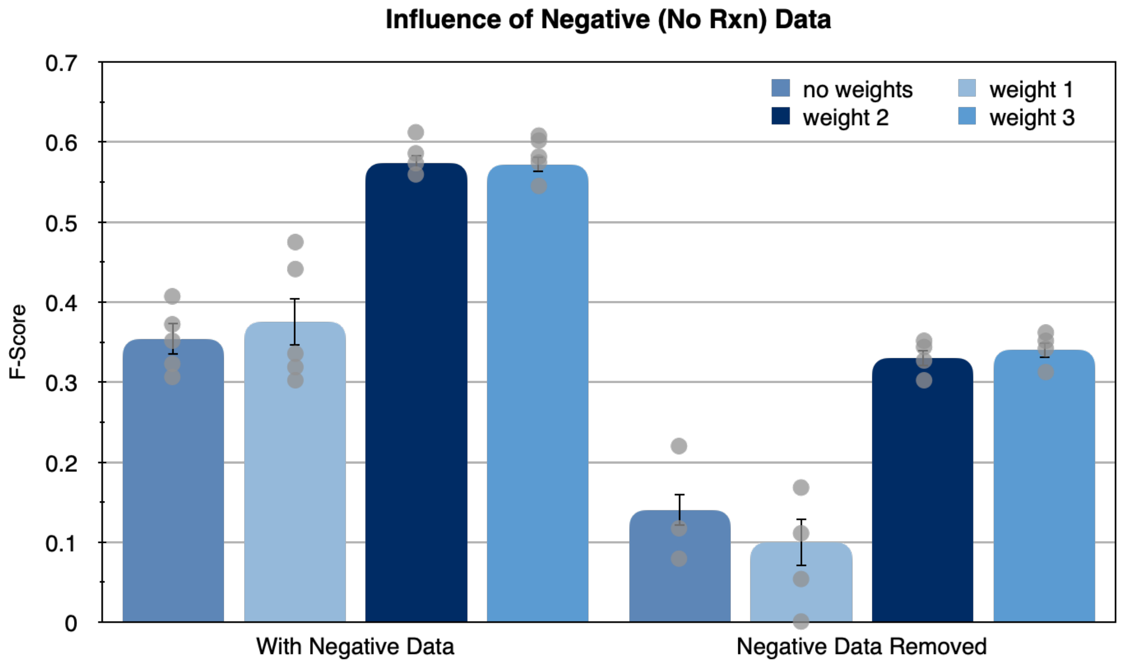

Figure S4: The effect of removing datapoints that saw no reaction (n=5). The bars in the bar charts represent the average with gray dots representing the individual data points (initializations with identical values are shown as a single point). Standard error bars shown. Source data for each bar chart can be found in source data excel file.

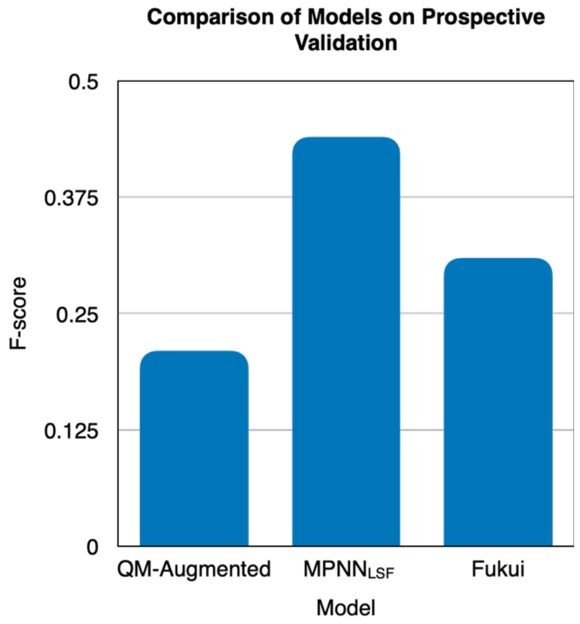

Figure S5: Comparison of $MPNN_{LSF}$ (best model on retrospective test set) to the Quantum Mechanics-augmented message passing neural network (QM-augmented; molecular dynamics simulations on atomic density representations) on the prospective test set (n=1).

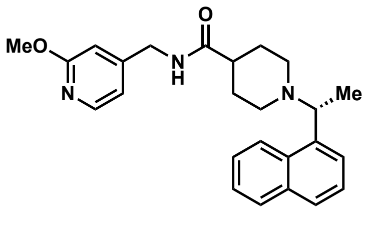

P450S1

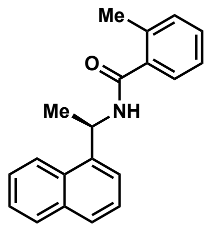

P450S2

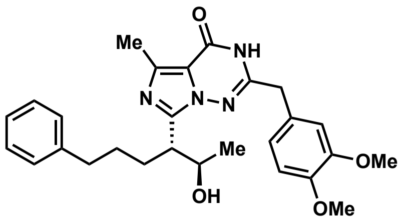

P450S3

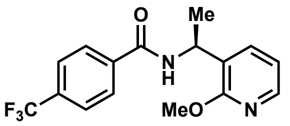

P450S4

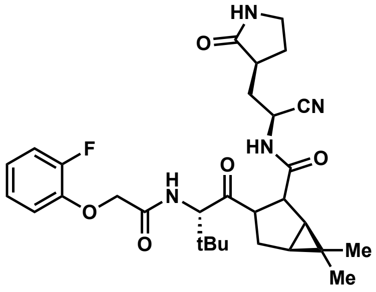

P450S5

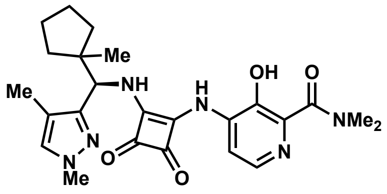

P450S6

Figure S6: Representative molecules in the P450-only test set.

Compound Characterizations:

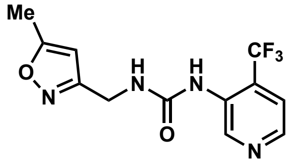

S1

Prepared according to General Minisci Functionalization with Baran Diversinates™ Procedure.

^{1}H NMR (600 MHz, DMSO-d$_6$): δ 9.16 (s, 1H), 8.47 (d, *J* = 2.9 Hz, 1H), 8.22 (s, 1H), 7.66 (d, *J* = 5.1 Hz, 1H), 7.52 (t, *J* = 5.8 Hz, 1H), 6.18 (s, 1H), 4.34 (d, *J* = 5.7 Hz, 2H), 2.39 (s, 3H).

HRMS: calcd for $C_{12}H_{11}F_3N_4O_2H^+$ ([M+H]) 301.0907, found 301.0917.

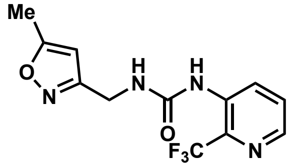

S2

Prepared according to General Minisci Functionalization with Baran Diversinates™ Procedure.

^{1}H NMR (600 MHz, DMSO-d$_6$): δ 8.42 (d, *J* = 8.5 Hz, 1H), 8.36 (d, *J* = 4.5 Hz, 1H), 8.20 (s, 1H), 7.65 (dd, *J* = 8.5, 4.5 Hz, 1H), 7.60 (t, *J* = 5.8 Hz, 1H), 6.17 (s, 1H), 4.33 (d, *J* = 5.7 Hz, 2H), 2.39 (s, 3H).

HRMS: calcd for $C_{12}H_{11}F_3N_4O_2H^+$ ([M+H]) 301.0907, found 301.0907.

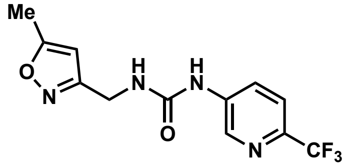

S3

Prepared according to General Minisci Functionalization with Baran Diversinates™ Procedure.

^{1}H NMR (600 MHz, DMSO-d$_6$): δ 9.37 (s, 1H), 8.69 (d, *J* = 2.5 Hz, 1H), 8.17 (dd, *J* = 8.5, 2.5 Hz, 1H), 7.77 (d, *J* = 8.7 Hz, 1H), 7.00 (t, *J* = 5.9 Hz, 1H), 6.17 (s, 1H), 4.33 (d, *J* = 5.8 Hz, 2H), 2.38 (s, 3H).

HRMS: calcd for $C_{12}H_{11}F_3N_4O_2H^+$ ([M+H]) 301.0907, found 301.0914.

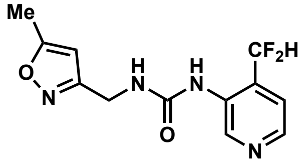

S4

Prepared according to General Minisci Functionalization with Baran Diversinates™ Procedure.

^{1}H NMR (600 MHz, DMSO-d$_6$): δ 8.96 (s, 1H), 8.43 (s, 1H), 8.41 (d, *J* = 5.0 Hz, 1H), 7.50 (d, *J* = 5.0 Hz, 1H), 7.26 (t, *J* = 5.9 Hz, 1H), 7.12 (t, *J* = 54.4 Hz, 1H), 6.18 (s, 1H), 4.33 (d, *J* = 5.8 Hz, 2H), 2.39 (s, 3H).

HRMS: calcd for $C_{12}H_{12}F_2N_4O_2H^+$ ([M+H]) 283.1001, found 283.1002.

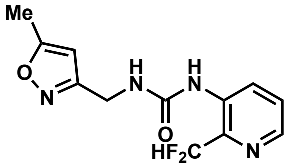

S5

Prepared according to General Minisci Functionalization with Baran Diversinates™ Procedure.

^{1}H NMR (600 MHz, DMSO-d$_6$): δ 8.33 (s, 1H), 8.32 – 8.29 (m, 2H), 7.51 (dd, *J* = 8.5, 4.6 Hz, 1H), 7.48 (t, *J* = 5.7 Hz, 1H), 7.00 (t, *J* = 53.8 Hz, 1H), 6.17 (s, 1H), 4.32 (d, *J* = 5.7 Hz, 2H), 2.39 (s, 3H).

HRMS: calcd for $C_{12}H_{12}F_2N_4O_2H^+$ ([M+H]) 283.1001, found 283.1008.

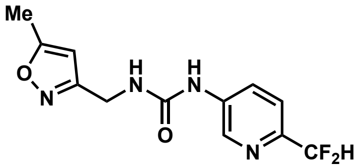

S6

Prepared according to General Minisci Functionalization with Baran Diversinates™ Procedure.

^{1}H NMR (600 MHz, DMSO-d$_6$): δ 9.19 (s, 1H), 8.64 (d, *J* = 2.5 Hz, 1H), 8.08 (dd, *J* = 8.7, 2.5 Hz, 1H), 7.58 (d, *J* = 8.6 Hz, 1H), 6.93 (t, *J* = 5.8 Hz, 1H), 6.85 (t, *J* = 55.3 Hz, 1H), 6.16 (s, 1H), 4.32 (d, *J* = 5.7 Hz, 2H), 2.38 (s, 3H).

HRMS: calcd for $C_{12}H_{12}F_2N_4O_2H^+$ ([M+H]) 283.1001, found 283.1003.

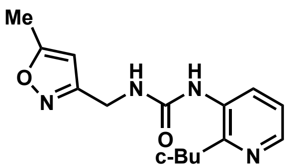

S7

Prepared according to General Minisci Functionalization with Molander BF_3K Salts Procedure.

^{1}H NMR (600 MHz, DMSO-d$_6$): δ 8.19 (d, *J* = 4.3 Hz, 1H), 8.12 (d, *J* = 8.2 Hz, 1H), 7.80 (s, 1H), 7.19 (t, *J* = 5.8 Hz, 1H), 7.16 (dd, *J* = 8.2, 4.8 Hz, 1H), 6.17 (s, 1H), 4.31 (d, *J* = 5.7 Hz,

2H), 3.76 (p, J = 8.5 Hz, 1H), 2.39 (s, 3H), 2.35 – 2.24 (m, 4H), 2.04 – 1.94 (m, 1H), 1.85 – 1.78 (m, 1H).

HRMS: calcd for $C_{15}H_{16}N_4O_2H^+$ ([M+H]) 287.1503, found 287.1511.

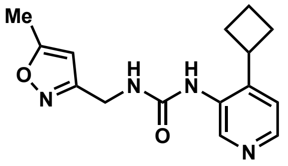

S8

Prepared according to General Minisci Functionalization with Molander BF_3K Salts Procedure.

^{1}H NMR (600 MHz, DMSO-d$_6$): δ 8.82 (s, 1H), 8.24 (s, 1H), 7.81 (s, 1H), 7.31 (s, 1H), 7.14 (t, J = 6.2 Hz, 1H), 6.17 (s, 1H), 4.31 (d, J = 5.8 Hz, 2H), 3.61 (p, J = 9.0, 8.5 Hz, 1H), 2.39 (s, 3H), 2.39 – 2.32 (m, 2H), 2.09 – 1.96 (m, 3H), 1.84 – 1.77 (m, 1H).

HRMS: calcd for $C_{15}H_{16}N_4O_2H^+$ ([M+H]) 287.1503, found 287.1506.

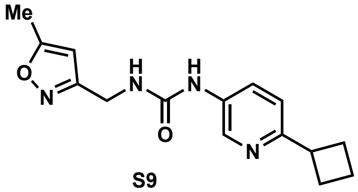

S9

Prepared according to General Minisci Functionalization with Molander BF_3K Salts Procedure.

^{1}H NMR (600 MHz, DMSO-d$_6$): δ 8.81 (s, 1H), 8.49 (s, 1H), 7.83 (d, J = 8.5 Hz, 1H), 7.16 (s, 1H), 6.76 (s, 1H), 6.15 (s, 1H), 4.30 (d, J = 5.9 Hz, 2H), 3.57 (p, J = 8.4, 7.9 Hz, 1H), 2.37 (s, 3H), 2.27 – 2.19 (m, 4H), 2.00 – 1.93 (m, 1H), 1.86 – 1.78 (m, 1H).

HRMS: calcd for $C_{15}H_{16}N_4O_2H^+$ ([M+H]) 287.1503, found 287.1496.

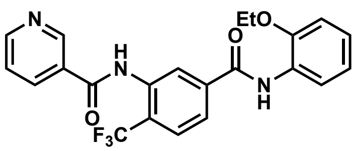

S10

Prepared according to General Minisci Functionalization with Baran Diversinates™ Procedure.

^{1}H NMR (600 MHz, DMSO-d$_6$): δ 10.57 (s, 1H), 9.76 (s, 1H), 9.13 (d, J = 2.3 Hz, 1H), 8.81 (dd, J = 4.9, 1.7 Hz, 1H), 8.31 (dt, J = 8.0, 2.0 Hz, 1H), 8.13 (s, 1H), 8.12 (d, J = 8.9 Hz, 1H), 8.01 (d, J = 8.2 Hz, 1H), 7.69 (d, J = 7.9 Hz, 1H), 7.62 (dd, J = 8.1, 4.6 Hz, 1H), 7.21 (t, J = 7.4 Hz, 1H), 7.11 (d, J = 8.4 Hz, 1H), 6.98 (t, J = 7.6 Hz, 1H), 4.11 (q, J = 7.0 Hz, 2H), 1.34 (t, J = 6.8 Hz, 3H).

HRMS: calcd for $C_{22}H_{18}F_3N_3O_3H^+$ ([M+H]) 430.1373, found 430.1366.

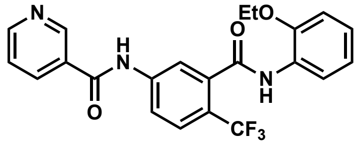

S11

Prepared according to General Minisci Functionalization with Baran Diversinates™ Procedure.

^{1}H NMR (600 MHz, DMSO-d$_6$): δ 10.90 (s, 1H), 9.64 (s, 1H), 9.15 (s, 1H), 8.81 (d, *J* = 4.8 Hz, 1H), 8.34 (dt, *J* = 8.1, 2.1 Hz, 1H), 8.13 (d, *J* = 2.2 Hz, 1H), 8.10 (d, *J* = 8.7 Hz, 1H), 7.85 (d, *J* = 8.7 Hz, 1H), 7.79 (d, *J* = 7.9 Hz, 1H), 7.62 (dd, *J* = 8.0, 4.7 Hz, 1H), 7.18 (t, *J* = 7.9 Hz, 1H), 7.09 (d, *J* = 8.2 Hz, 1H), 6.97 (t, *J* = 7.6 Hz, 1H), 4.09 (q, *J* = 6.9 Hz, 2H), 1.35 (t, *J* = 7.0 Hz, 3H).

HRMS: calcd for $C_{22}H_{18}F_3N_3O_3H^+$ ([M+H]) 430.1373, found 430.1375.

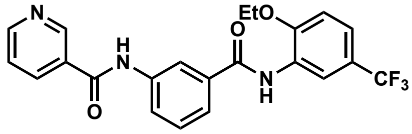

S12

Prepared according to General Minisci Functionalization with Baran Diversinates™ Procedure.

^{1}H NMR (600 MHz, DMSO-d$_6$): δ 10.68 (s, 1H), 9.55 (s, 1H), 9.15 (d, *J* = 2.3 Hz, 1H), 8.79 (dd, *J* = 4.7, 1.7 Hz, 1H), 8.41 (s, 1H), 8.34 (dt, *J* = 8.0, 2.0 Hz, 1H), 8.28 (d, *J* = 2.4 Hz, 1H), 8.01 (dd, *J* = 8.0, 2.2 Hz, 1H), 7.73 (d, *J* = 7.7 Hz, 1H), 7.60 (dd, *J* = 8.0, 4.8 Hz, 1H), 7.57 (t, *J* = 8.1 Hz, 1H), 7.54 (dd, *J* = 8.8, 2.8 Hz, 1H), 7.30 (d, *J* = 8.6 Hz, 1H), 4.24 (q, *J* = 7.0 Hz, 2H), 1.42 (t, *J* = 7.0 Hz, 3H).

HRMS: calcd for $C_{22}H_{18}F_3N_3O_3H^+$ ([M+H]) 430.1373, found 430.1369.

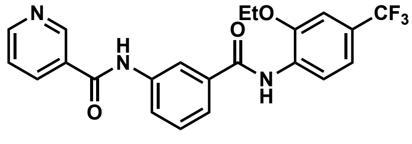

S13

Prepared according to General Minisci Functionalization with Baran Diversinates™ Procedure.

^{1}H NMR (600 MHz, DMSO-d$_6$): δ 10.68 (s, 1H), 9.52 (s, 1H), 9.15 (s, 1H), 8.79 (d, *J* = 4.9 Hz, 1H), 8.42 (s, 1H), 8.34 (dt, *J* = 8.1, 2.0 Hz, 1H), 8.19 (d, *J* = 8.2 Hz, 1H), 8.03 (dd, *J* = 8.0, 2.2 Hz, 1H), 7.73 (d, *J* = 7.7 Hz, 1H), 7.60 (dd, *J* = 8.0, 4.9 Hz, 1H), 7.57 (t, *J* = 7.9 Hz, 1H), 7.39 (s, 1H), 7.37 (d, *J* = 9.1 Hz, 1H), 4.24 (q, *J* = 6.9 Hz, 2H), 1.42 (t, *J* = 6.9 Hz, 3H).

HRMS: calcd for $C_{22}H_{18}F_3N_3O_3H^+$ ([M+H]) 430.1373, found 430.1373.

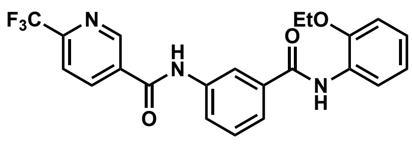

S14

Prepared according to General Minisci Functionalization with Baran Diversinates™ Procedure.

^{1}H NMR (600 MHz, DMSO-d$_6$): δ 10.85 (s, 1H), 9.35 (s, 1H), 9.28 (s, 1H), 8.61 (d, *J* = 8.0 Hz, 1H), 8.37 (s, 1H), 8.13 (d, *J* = 8.1 Hz, 1H), 8.01 (d, *J* = 7.8 Hz, 1H), 7.87 (d, *J* = 7.9 Hz, 1H), 7.74 (d, *J* = 8.0 Hz, 1H), 7.57 (t, *J* = 7.9 Hz, 1H), 7.16 (t, *J* = 8.1 Hz, 1H), 7.10 (d, *J* = 8.2 Hz, 1H), 6.97 (t, *J* = 7.7 Hz, 1H), 4.12 (q, *J* = 7.0 Hz, 2H), 1.38 (t, *J* = 6.7 Hz, 3H).

HRMS: calcd for $C_{22}H_{18}F_3N_3O_3H^+$ ([M+H]) 430.1373, found 430.1367.

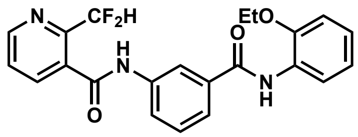

S15

Prepared according to General Minisci Functionalization with Baran Diversinates™ Procedure.

^{1}H NMR (600 MHz, DMSO-d$_6$): δ 10.87 (s, 1H), 9.33 (s, 1H), 8.85 (d, *J* = 4.7 Hz, 1H), 8.35 (s, 1H), 8.23 (d, *J* = 7.8 Hz, 1H), 7.92 – 7.87 (m, 2H), 7.75 (dd, *J* = 7.9, 5.0 Hz, 1H), 7.73 (d, *J* = 7.8 Hz, 1H), 7.55 (t, *J* = 7.9 Hz, 1H), 7.22 (t, *J* = 53.9 Hz, 1H), 7.16 (t, *J* = 7.8 Hz, 1H), 7.10 (d, *J* = 8.2 Hz, 1H), 6.98 (t, *J* = 7.6 Hz, 1H), 4.13 (q, *J* = 6.9 Hz, 2H), 1.39 (t, *J* = 6.9 Hz, 3H).

HRMS: calcd for $C_{22}H_{19}F_2N_3O_3H^+$ ([M+H]) 412.1467, found 412.1472.

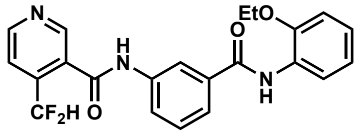

S16

Prepared according to General Minisci Functionalization with Baran Diversinates™ Procedure.

^{1}H NMR (600 MHz, DMSO-d$_6$): δ 10.96 (s, 1H), 9.33 (s, 1H), 9.06 (s, 1H), 8.94 (d, *J* = 5.1 Hz, 1H), 8.37 (s, 1H), 7.93 – 7.88 (m, 2H), 7.78 (d, *J* = 5.1 Hz, 1H), 7.74 (d, *J* = 7.7 Hz, 1H), 7.56 (t, *J* = 7.9 Hz, 1H), 7.42 (t, *J* = 54.6 Hz, 1H), 7.16 (t, *J* = 7.8 Hz, 1H), 7.11 (d, *J* = 8.1 Hz, 1H), 6.98 (t, *J* = 7.6 Hz, 1H), 4.13 (q, *J* = 7.0 Hz, 2H), 1.39 (t, *J* = 6.9 Hz, 3H).

HRMS: calcd for $C_{22}H_{19}F_2N_3O_3H^+$ ([M+H]) 412.1467, found 412.1473.

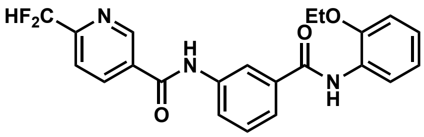

S17

Prepared according to General Minisci Functionalization with Baran Diversinates™ Procedure.

^{1}H NMR (600 MHz, DMSO-d$_6$): δ 10.78 (s, 1H), 9.35 (s, 1H), 9.23 (s, 1H), 8.53 (dd, *J* = 8.1, 2.3 Hz, 1H), 8.38 (s, 1H), 8.02 (d, *J* = 8.0 Hz, 1H), 7.91 (d, *J* = 8.1 Hz, 1H), 7.88 (d, *J* = 7.9 Hz, 1H), 7.74 (d, *J* = 7.7 Hz, 1H), 7.57 (t, *J* = 7.9 Hz, 1H), 7.16 (d, *J* = 7.7 Hz, 1H), 7.11 (d, *J* = 8.3 Hz, 1H), 7.09 (t, *J* = 55.1 Hz, 1H), 6.98 (d, *J* = 7.6 Hz, 1H), 4.13 (q, *J* = 6.9 Hz, 2H), 1.39 (t, *J* = 6.9 Hz, 3H).

HRMS: calcd for $C_{22}H_{19}F_2N_3O_3H^+$ ([M+H]) 412.1467, found 412.1467.

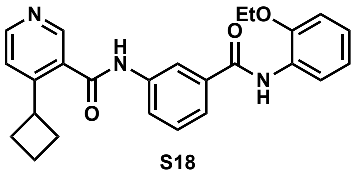

S18

Prepared according to General Minisci Functionalization with Molander BF_3K Salts Procedure.

^{1}H NMR (600 MHz, DMSO-d$_6$): δ 10.75 (s, 1H), 9.31 (s, 1H), 8.80 – 8.64 (m, 2H), 8.36 (s, 1H), 7.91 (d, *J* = 7.9 Hz, 1H), 7.87 (dd, *J* = 8.0, 2.2 Hz, 1H), 7.71 (d, *J* = 7.7 Hz, 1H), 7.59 (d, *J* = 5.1 Hz, 1H), 7.54 (t, *J* = 7.9 Hz, 1H), 7.16 (td, *J* = 7.8, 1.7 Hz, 1H), 7.10 (d, *J* = 8.0 Hz, 1H), 6.98 (t, *J* = 7.5 Hz, 1H), 4.13 (q, *J* = 6.9 Hz, 2H), 3.92 (p, *J* = 8.8 Hz, 1H), 2.34 – 2.25 (m, 2H), 2.22 – 2.11 (m, 2H), 2.05 – 1.93 (m, 1H), 1.84 – 1.75 (m, 1H), 1.39 (t, *J* = 7.0 Hz, 3H).

HRMS: calcd for $C_{25}H_{25}N_3O_3H^+$ ([M+H]) 416.1969, found 416.1968.

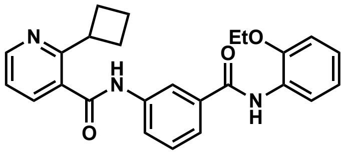

S19

Prepared according to General Minisci Functionalization with Molander BF_3K Salts Procedure.

^{1}H NMR (600 MHz, DMSO-d$_6$): δ 10.66 (s, 1H), 9.31 (s, 1H), 8.70 (dd, *J* = 5.0, 1.7 Hz, 1H), 8.37 (s, 1H), 7.93 – 7.88 (m, 2H), 7.87 (d, *J* = 7.8 Hz, 1H), 7.70 (d, *J* = 7.7 Hz, 1H), 7.53 (t, *J* = 7.9 Hz, 1H), 7.40 (dd, *J* = 7.7, 4.9 Hz, 1H), 7.16 (t, *J* = 7.2 Hz, 1H), 7.10 (d, *J* = 8.1 Hz, 1H), 6.98 (t, *J* = 7.6 Hz, 1H), 4.13 (q, *J* = 6.9 Hz, 2H), 3.98 (p, *J* = 8.6 Hz, 1H), 2.45 – 2.34 (m, 2H), 2.26 – 2.16 (m, 2H), 2.03 – 1.91 (m, 1H), 1.84 – 1.75 (m, 1H), 1.39 (t, *J* = 6.9 Hz, 3H).

HRMS: calcd for $C_{25}H_{25}N_3O_3H^+$ ([M+H]) 416.1969, found 416.1960.

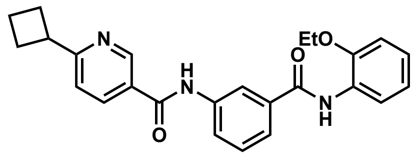

S20

Prepared according to General Minisci Functionalization with Molander BF_3K Salts Procedure.

^{1}H NMR (600 MHz, DMSO-d$_6$): δ 10.62 (s, 1H), 9.33 (s, 1H), 9.12 (d, *J* = 2.3 Hz, 1H), 8.40 – 8.32 (m, 2H), 8.01 (dd, *J* = 8.1, 2.2 Hz, 1H), 7.89 (dd, *J* = 7.9, 1.6 Hz, 1H), 7.72 (d, *J* = 7.7 Hz, 1H), 7.60 – 7.50 (m, 2H), 7.16 (td, *J* = 7.8, 1.7 Hz, 1H), 7.10 (d, *J* = 8.1 Hz, 1H), 6.98 (t, *J* = 7.5 Hz, 1H), 4.13 (q, *J* = 6.9 Hz, 2H), 3.79 (p, *J* = 8.6 Hz, 1H), 2.41 – 2.28 (m, 4H), 2.11 – 2.00 (m, 1H), 1.94 – 1.85 (m, 1H), 1.39 (t, *J* = 7.0 Hz, 3H).

HRMS: calcd for $C_{25}H_{25}N_3O_3H^+$ ([M+H]) 416.1969, found 416.1971.

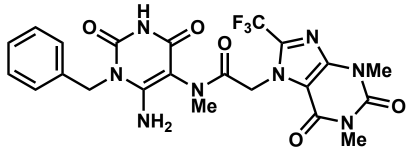

S21

Prepared according to General Minisci Functionalization with Baran Diversinates™ Procedure.

^{1}H NMR (600 MHz, DMSO-d$_6$): δ 11.01 (s, 1H), 7.35 (t, *J* = 7.5 Hz, 2H), 7.30 – 7.21 (m, 5H), 5.16 (q, *J* = 17.5, 17.0 Hz, 2H), 5.05 (d, *J* = 16.5 Hz, 1H), 4.96 (d, *J* = 16.6 Hz, 1H), 3.46 (s, 3H), 3.23 (s, 3H), 2.92 (s, 3H).

HRMS: calcd for $C_{22}H_{21}F_3N_8O_5H^+$ ([M+H]) 535.1660, found 535.1666.

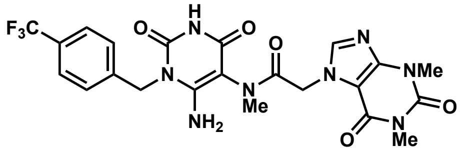

S22

Prepared according to General Minisci Functionalization with Baran Diversinates™ Procedure.

^{1}H NMR (600 MHz, DMSO-d$_6$): δ 11.07 (s, 1H), 7.99 (s, 1H), 7.73 (d, *J* = 8.1 Hz, 2H), 7.47 (d, *J* = 8.0 Hz, 2H), 5.23 (q, *J* = 17.7 Hz, 2H), 5.08 (d, *J* = 16.6 Hz, 1H), 4.95 (d, *J* = 16.6 Hz, 1H), 3.45 (s, 3H), 3.20 (s, 3H), 2.93 (s, 3H).

HRMS: calcd for $C_{22}H_{21}F_3N_8O_5H^+$ ([M+H]) 535.1660, found 535.1655.

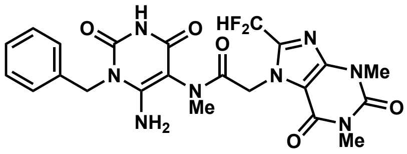

S23

Prepared according to General Minisci Functionalization with Baran Diversinates™ Procedure.

^{1}H NMR (600 MHz, DMSO-d$_6$): δ 11.04 (s, 1H), 7.35 (t, *J* = 7.5 Hz, 2H), 7.30 – 7.23 (m, 5H), 7.12 (t, *J* = 51.7 Hz, 1H), 5.25 (s, 2H), 5.15 (q, *J* = 17.0 Hz, 2H), 3.46 (s, 3H), 3.22 (s, 3H), 2.92 (s, 3H).

HRMS: calcd for $C_{22}H_{22}F_2N_8O_5H^+$ ([M+H]) 517.1754, found 517.1744.

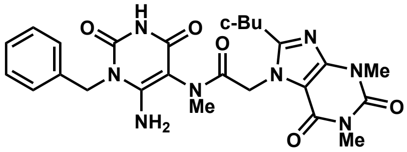

S24

Prepared according to General Minisci Functionalization with Molander BF_3K Salts Procedure.

^{1}H NMR (600 MHz, DMSO-d$_6$): δ 11.02 (s, 1H), 7.35 (t, *J* = 7.5 Hz, 2H), 7.29 – 7.23 (m, 5H), 5.16 (s, 2H), 4.97 (d, *J* = 17.2 Hz, 1H), 4.85 (d, *J* = 17.0 Hz, 1H), 3.55 (p, *J* = 8.6 Hz, 1H), 3.46 (s, 3H), 3.19 (s, 3H), 2.91 (s, 3H), 2.45 – 2.36 (m, 1H), 2.28 – 2.17 (m, 3H), 2.00 – 1.89 (m, 1H), 1.87 – 1.79 (m, 1H).

HRMS: calcd for $C_{25}H_{28}N_8O_5H^+$ ([M+H]) 521.2255, found 521.2263.

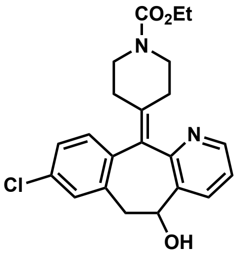

S25

Prepared according to Liver Microsomes and Recombinant CYP Enzyme Screen Procedure and Biomimetic Metalloporphyrin Oxidation Screen Procedure.

^{1}H NMR (600 MHz, DMSO-d$_6$): δ 8.42 − 8.36 (m, 1H), 7.88 − 7.76 (m, 1H), 7.32 − 7.19 (m, 3H), 7.14 − 7.07 (m, 1H), 6.11 − 5.55 (m, br, 1H), 5.19 − 5.17 (m, 1H), 4.08 − 4.00 (m, 2H), 3.70 − 3.55 (m, 2H), 3.50 − 3.45 (m, 1H), 3.27 − 3.11 (m, 2H), 3.07 − 2.74 (m, 1H), 2.43 − 2.01 (m, 4H), 1.21 − 1.14 (m, 3H).

HRMS: calcd for $C_{22}H_{23}ClN_2O_3H^+$ ([M+H]) 399.1475, found 399.1474.

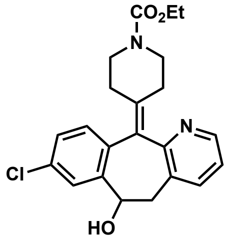

S26

Prepared according to Liver Microsomes and Recombinant CYP Enzyme Screen Procedure and Biomimetic Metalloporphyrin Oxidation Screen Procedure.

^{1}H NMR (600 MHz, DMSO-d$_6$): δ 8.38 − 8.30 (m, 1H), 7.77 − 7.43 (m, 2H), 7.27 (d, *J* = 8.0 Hz, 1H), 7.23 − 7.17 (m, 1H), 7.13 − 7.06 (m, 1H), 5.29 − 4.56 (m, 1H), 4.04 (q, *J* = 7.1 Hz, 2H), 3.76 − 3.56 (m, 2H), 3.50 − 3.33 (m, 1H), 3.27 − 3.12 (m, 2H), 3.06 − 2.73 (m, 1H), 2.46 − 2.35 (m, 1H), 2.28 − 2.16 (m, 2H), 2.14 − 1.99 (m, 1H), 1.17 (t, *J* = 7.1 Hz, 3H).

HRMS: calcd for $C_{22}H_{23}ClN_2O_3H^+$ ([M+H]) 399.1475, found 399.1475.

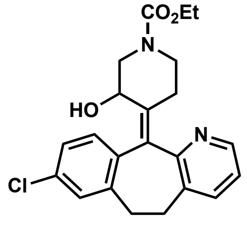

S27

Prepared according to Liver Microsomes and Recombinant CYP Enzyme Screen Procedure and Biomimetic Metalloporphyrin Oxidation Screen Procedure.

^{1}H NMR (600 MHz, DMSO-d$_6$): δ 8.35 (d, *J* = 4.7 Hz, 1H), 7.64 − 7.54 (m, 1H), 7.41 − 7.04 (m, 4H), 5.38 − 4.82 (m, 1H), 4.42 − 3.86 (m, 7H), 3.38 − 3.24 (m, 2H), 2.89 − 2.73 (m, 2H), 2.58 − 2.12 (m, 2H), 1.20 − 1.10 (m, 3H).

HRMS: calcd for $C_{22}H_{23}ClN_2O_3H^+$ ([M+H]) 399.1475, found 399.1475.

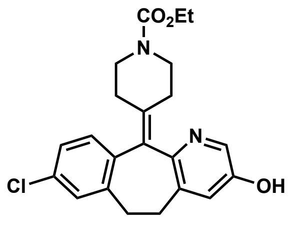

S28

Prepared according to Biomimetic Metalloporphyrin Oxidation Screen Procedure.

^{1}H NMR (600 MHz, DMSO-d$_6$): δ 7.90 (d, *J* = 2.6 Hz, 1H), 7.29 (d, *J* = 2.3 Hz, 1H), 7.19 (dd, *J* = 8.1, 2.3 Hz, 1H), 7.05 (d, *J* = 8.1 Hz, 1H), 6.93 (d, *J* = 2.7 Hz, 1H), 4.04 (q, *J* = 7.1 Hz, 2H), 3.65 − 3.55 (m, 2H), 3.37 − 3.11 (m, 4H), 2.83 − 2.68 (m, 2H), 2.36 − 2.29 (m, 1H), 2.28 − 2.21 (m, 1H), 2.21 − 2.10 (m, 2H), 1.17 (t, *J* = 7.1 Hz, 3H)

HRMS: calcd for $C_{22}H_{23}ClN_2O_3H^+$ ([M+H]) 399.1475, found 399.1478.

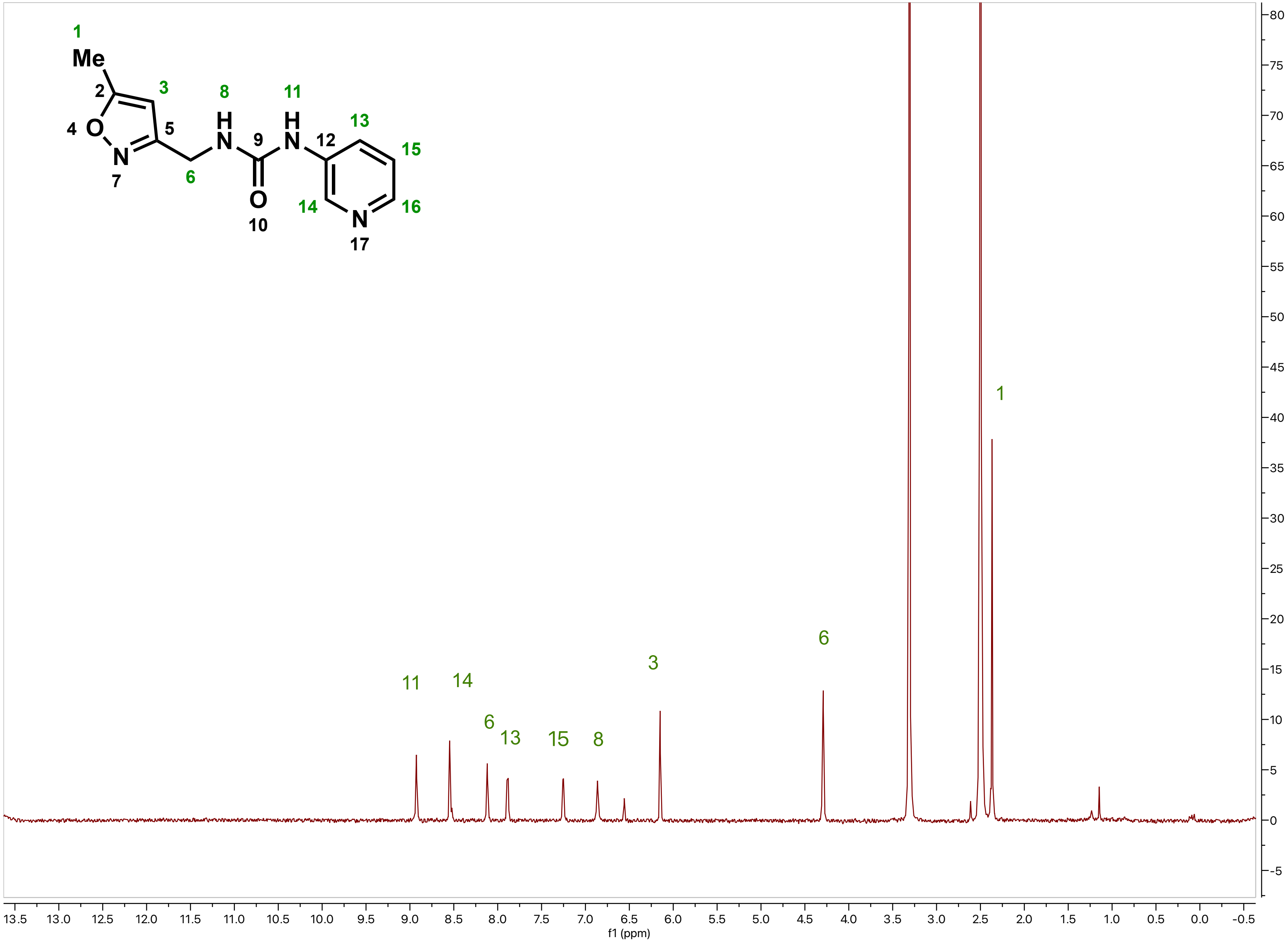

Figure S7: 600 MHz ^{1}H NMR spectrum for compound **6** in DMSO-d_6. Green numbered atoms are atoms with a potential ^{1}H NMR signal. Green numbered peaks are mapped to their corresponding atom number.

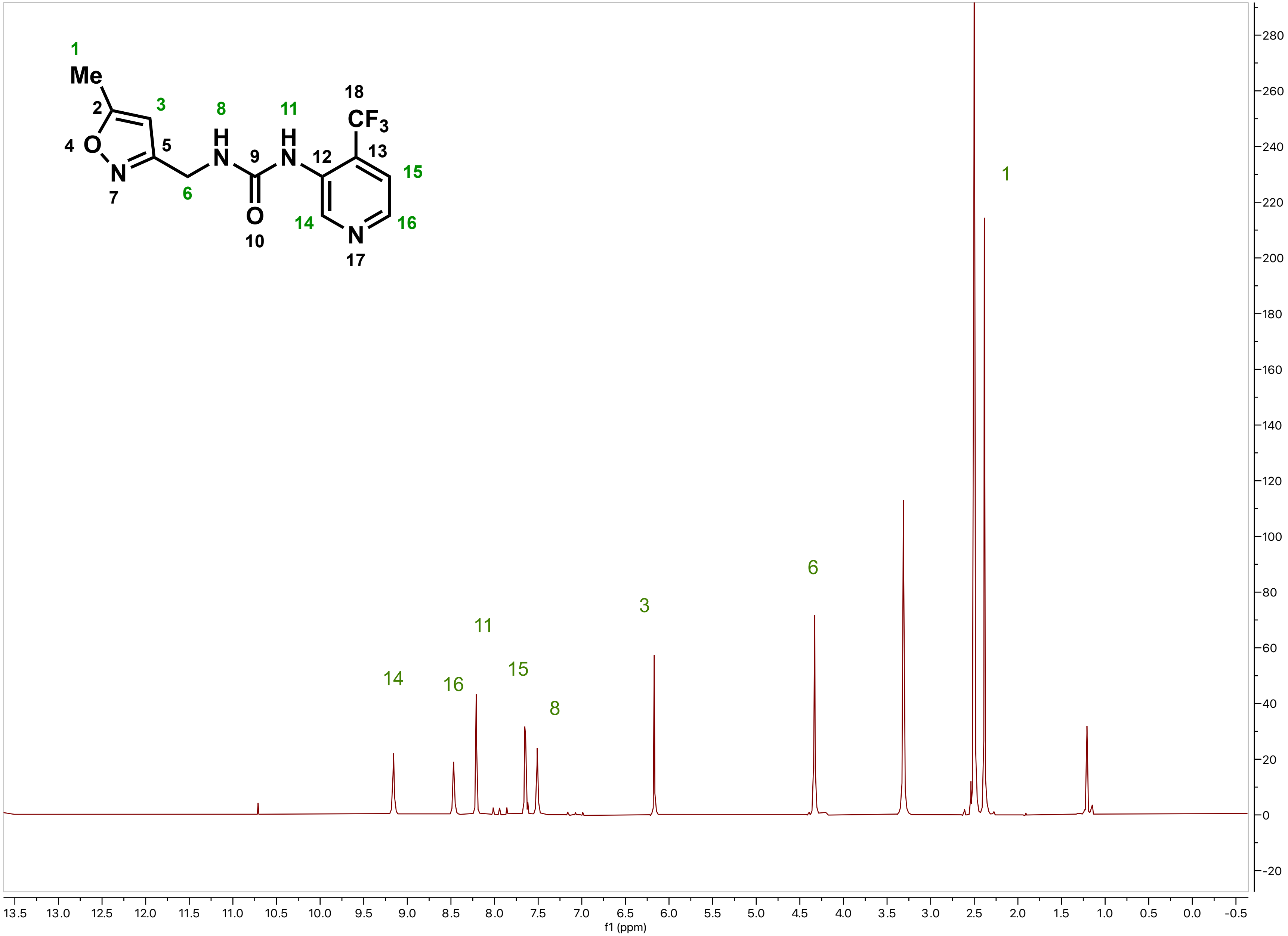

Figure S8: 600 MHz ^{1}H NMR spectrum for compound **S1** in DMSO-d$_6$. Green numbered atoms are atoms with a potential ^{1}H NMR signal. Green numbered peaks are mapped to their corresponding atom number.

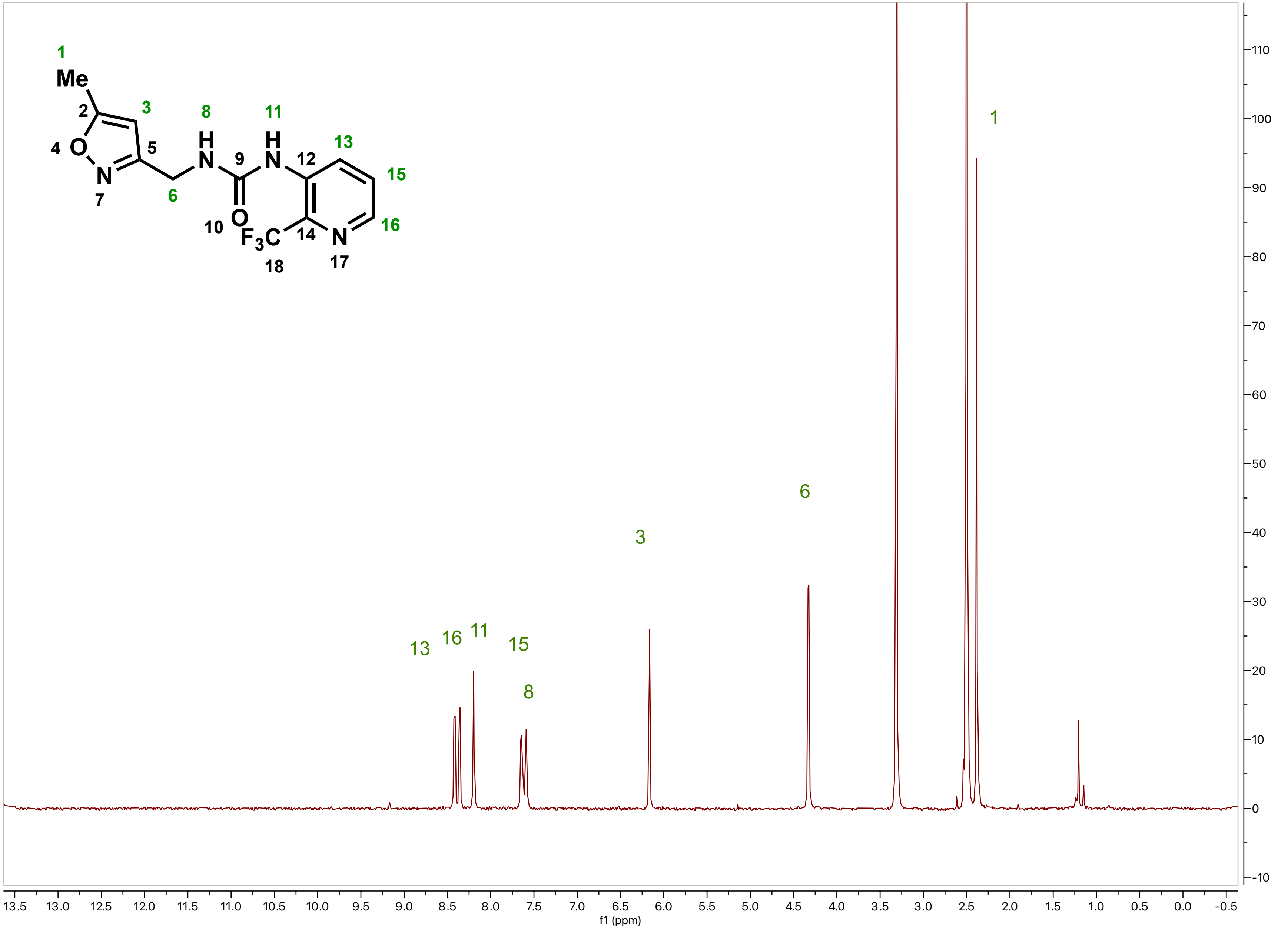

Figure S9: 600 MHz ^{1}H NMR spectrum for compound **S2** in DMSO-d_6. Green numbered atoms are atoms with a potential ^{1}H NMR signal. Green numbered peaks are mapped to their corresponding atom number.

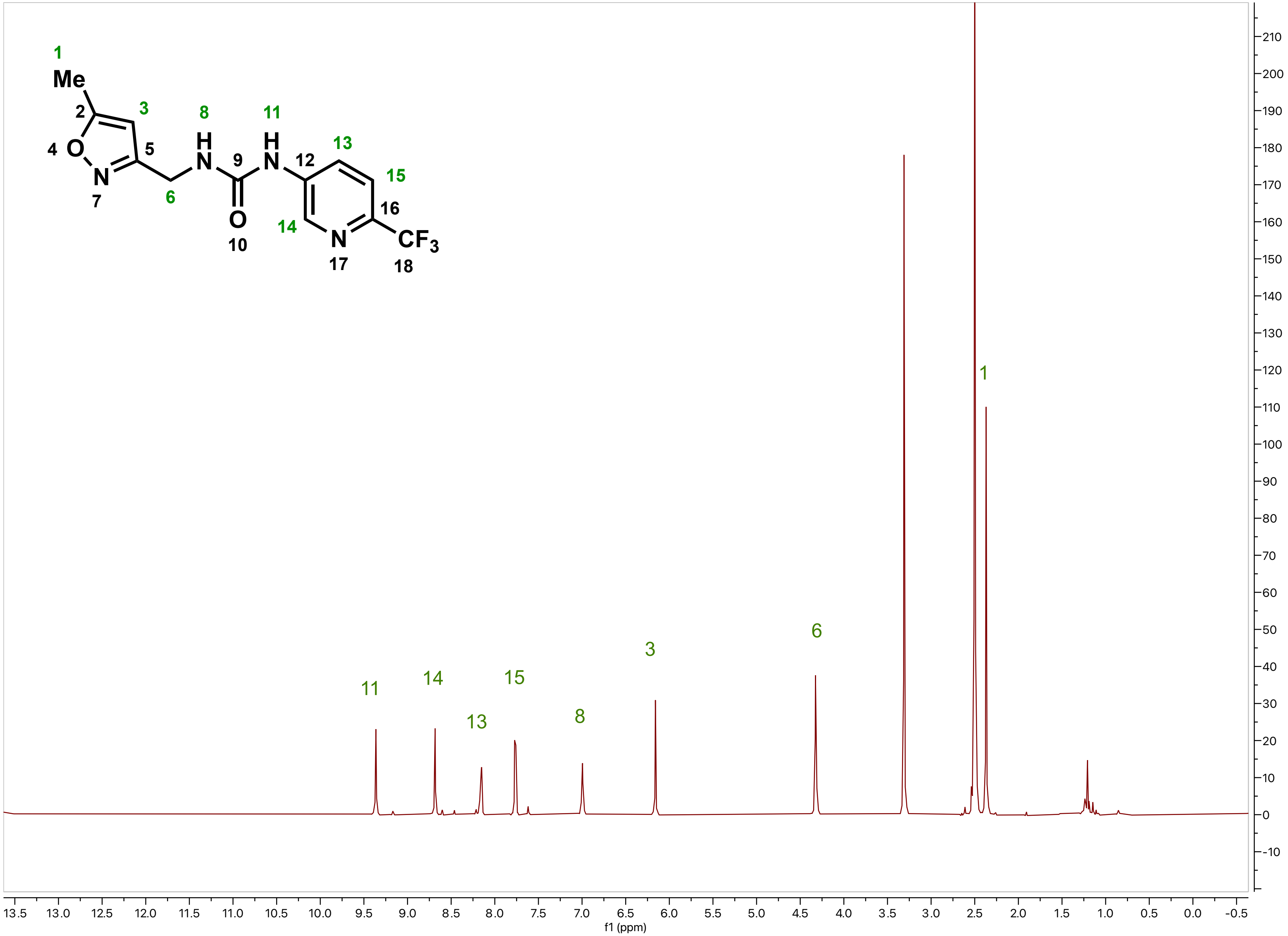

Figure S10: 600 MHz ^{1}H NMR spectrum for compound **S3** in DMSO-d_6. Green numbered atoms are atoms with a potential ^{1}H NMR signal. Green numbered peaks are mapped to their corresponding atom number.

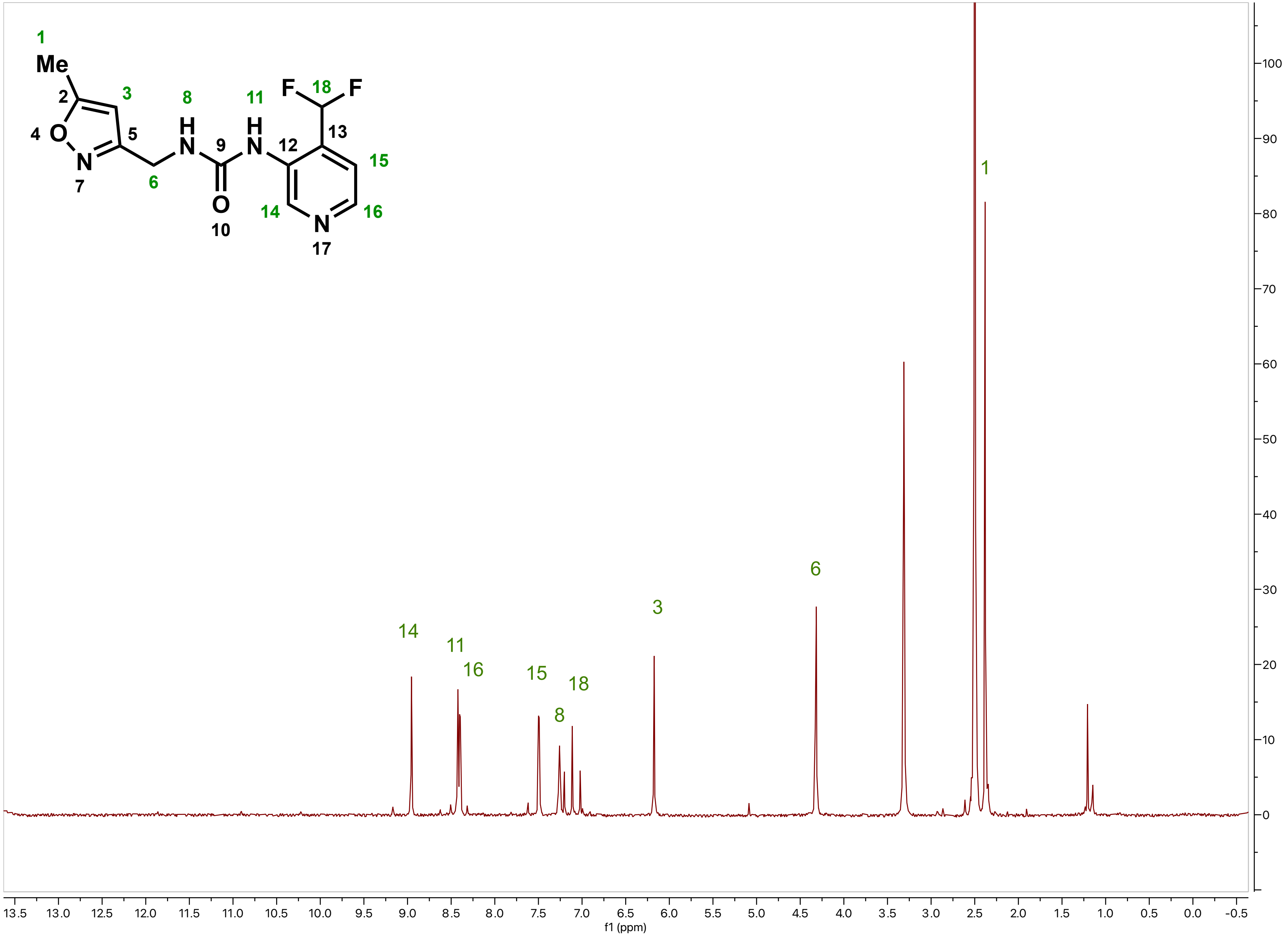

Figure S11: 600 MHz ^{1}H NMR spectrum for compound **S4** in DMSO-d$_6$. Green numbered atoms are atoms with a potential ^{1}H NMR signal. Green numbered peaks are mapped to their corresponding atom number.

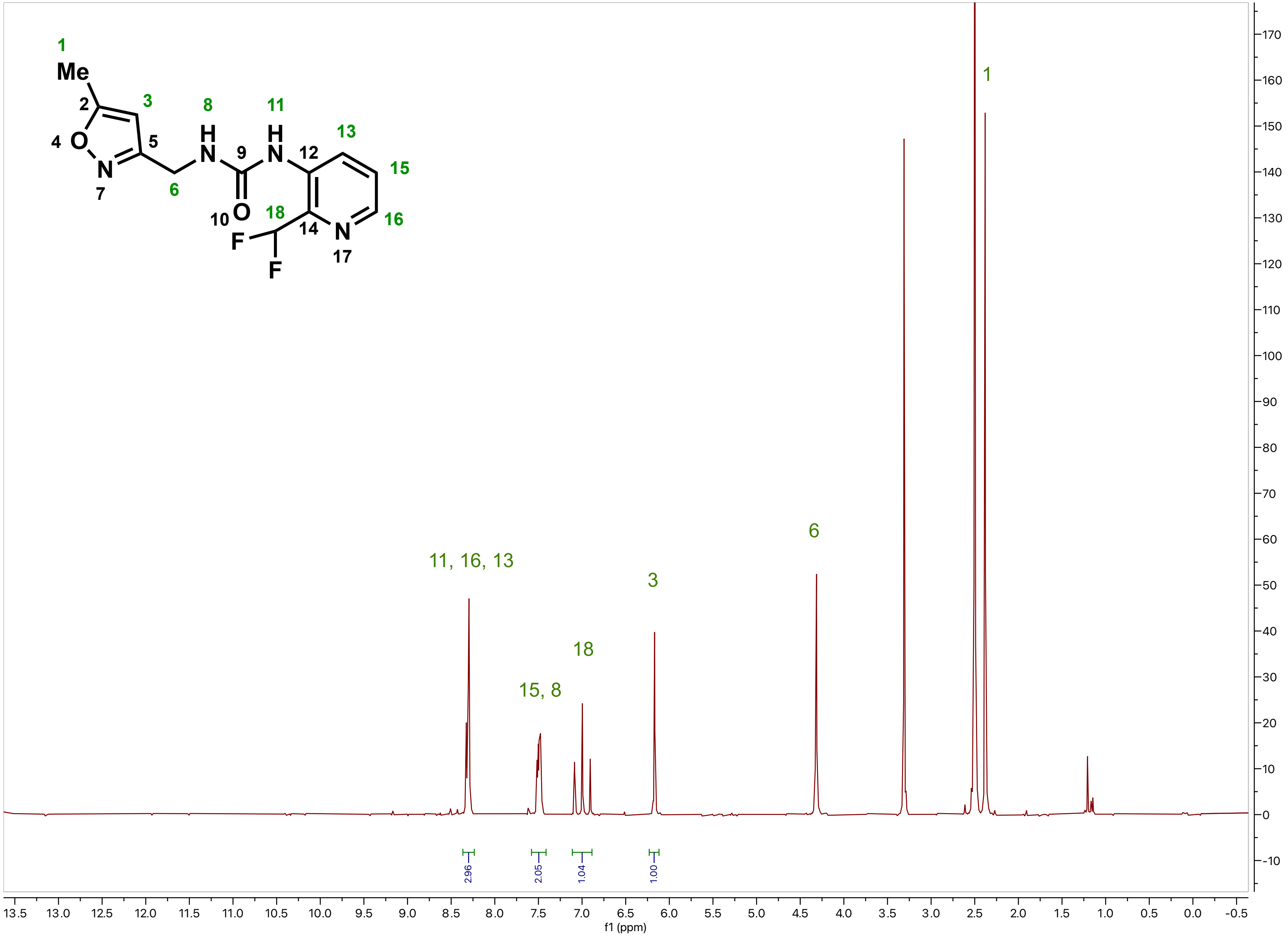

Figure S12: 600 MHz ^{1}H NMR spectrum for compound **S5** in DMSO-d_6. Green numbered atoms are atoms with a potential ^{1}H NMR signal. Green numbered peaks are mapped to their corresponding atom number.

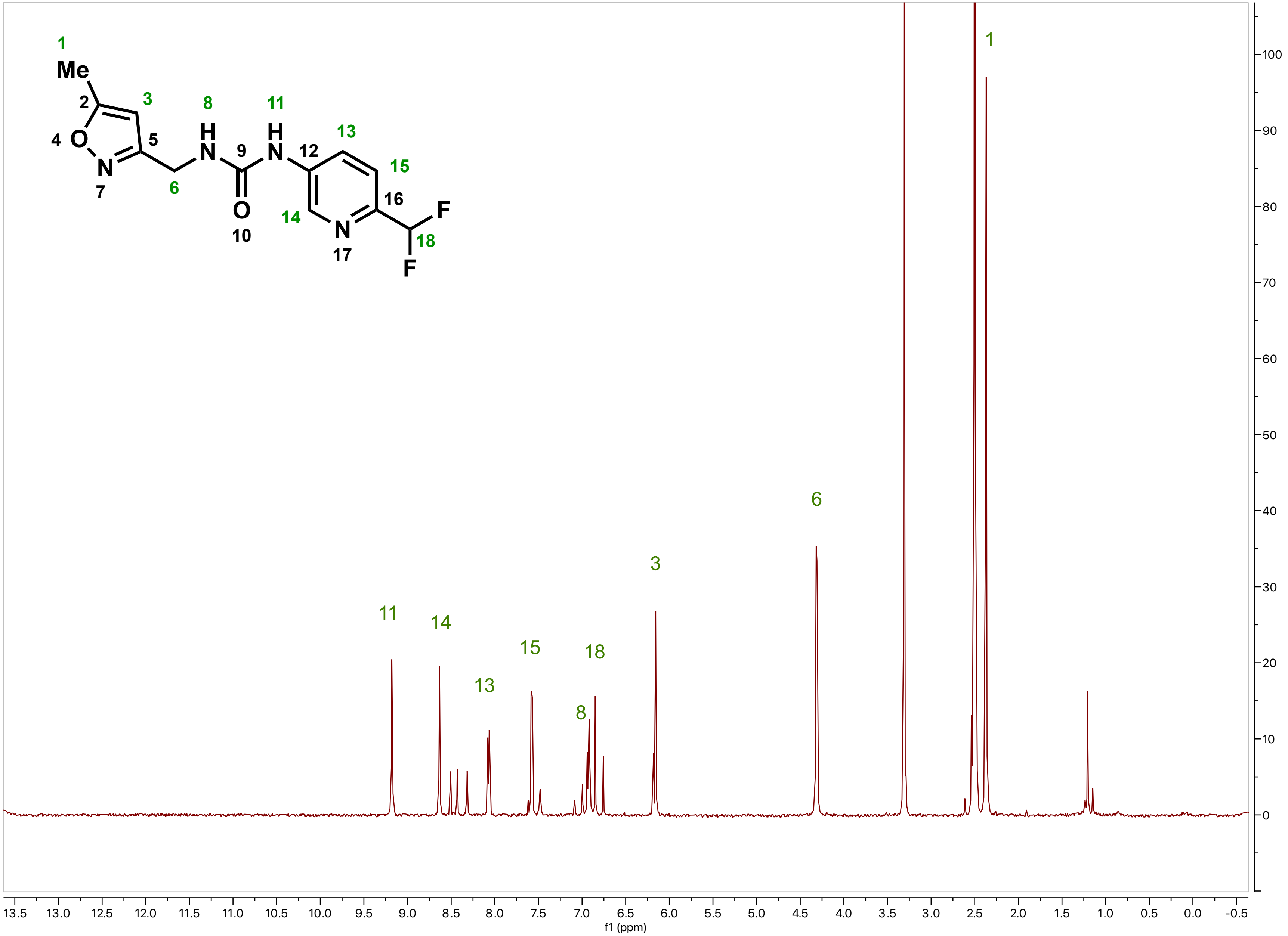

Figure S13: 600 MHz ^{1}H NMR spectrum for compound **S6** in DMSO-d_6. Green numbered atoms are atoms with a potential ^{1}H NMR signal. Green numbered peaks are mapped to their corresponding atom number.

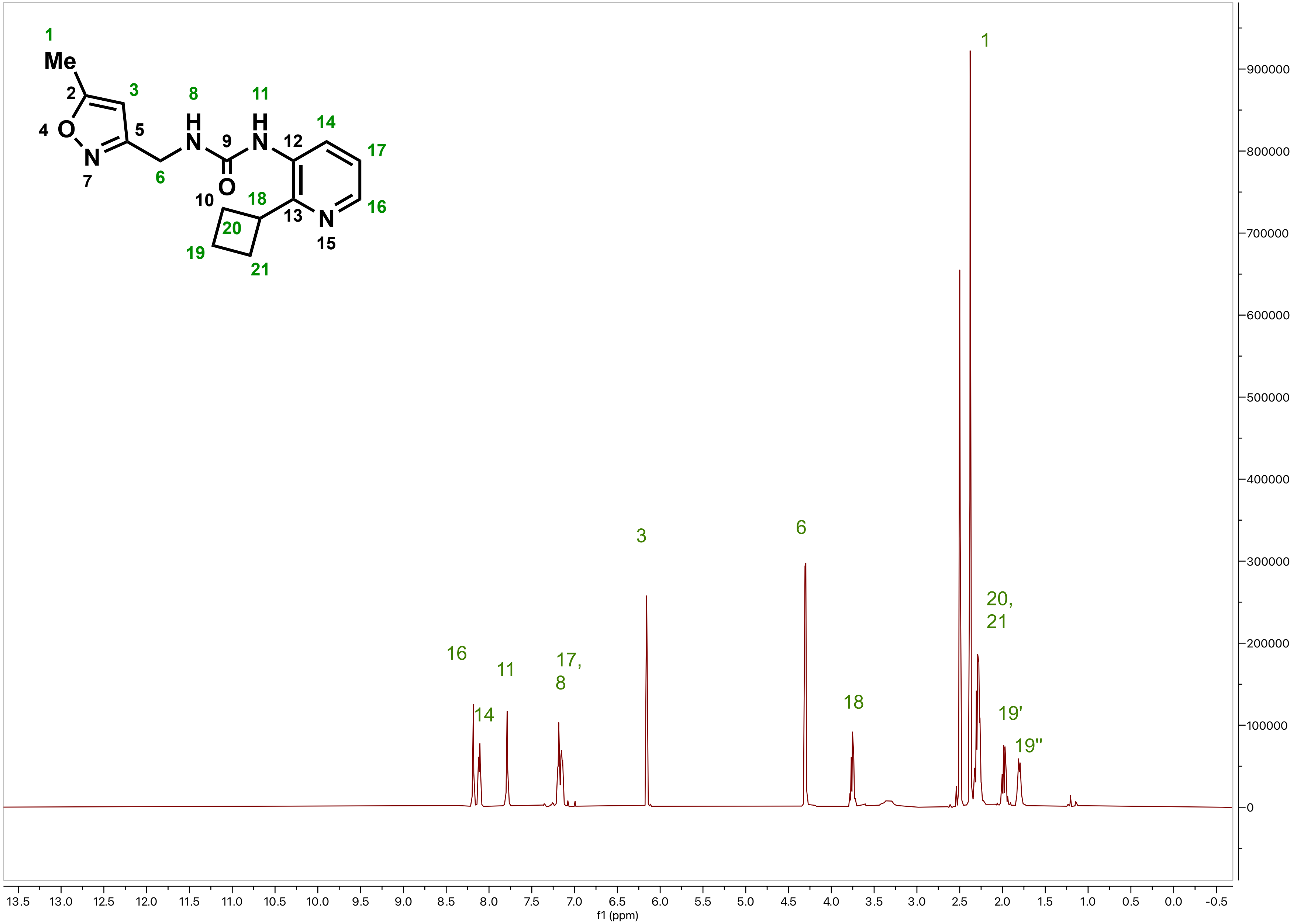

Figure S14: 600 MHz ^{1}H NMR spectrum for compound **S7** in DMSO-d$_6$. Green numbered atoms are atoms with a potential ^{1}H NMR signal. Green numbered peaks are mapped to their corresponding atom number.

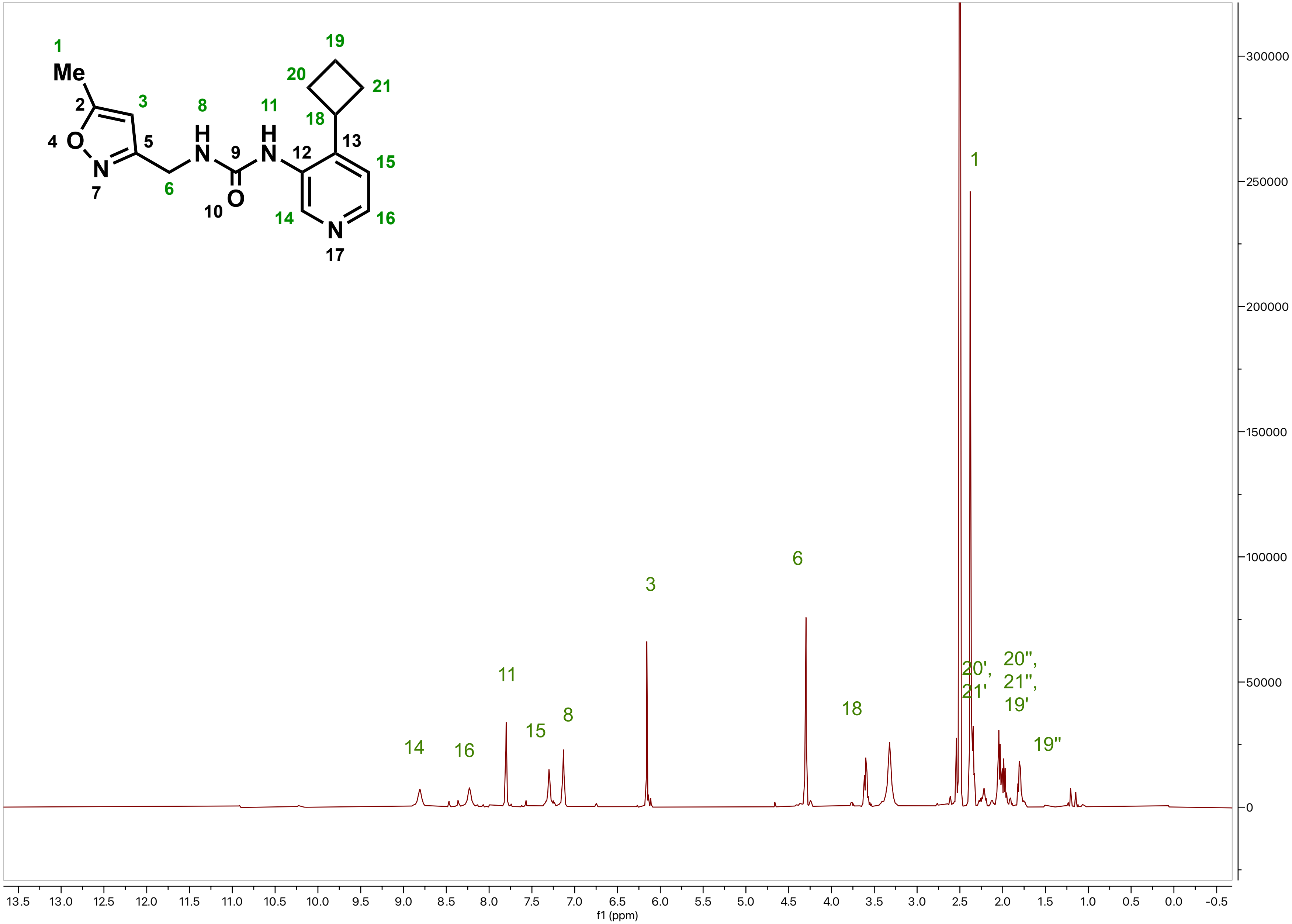

Figure S15: 600 MHz ^{1}H NMR spectrum for compound **S8** in DMSO-d_6. Green numbered atoms are atoms with a potential ^{1}H NMR signal. Green numbered peaks are mapped to their corresponding atom number.

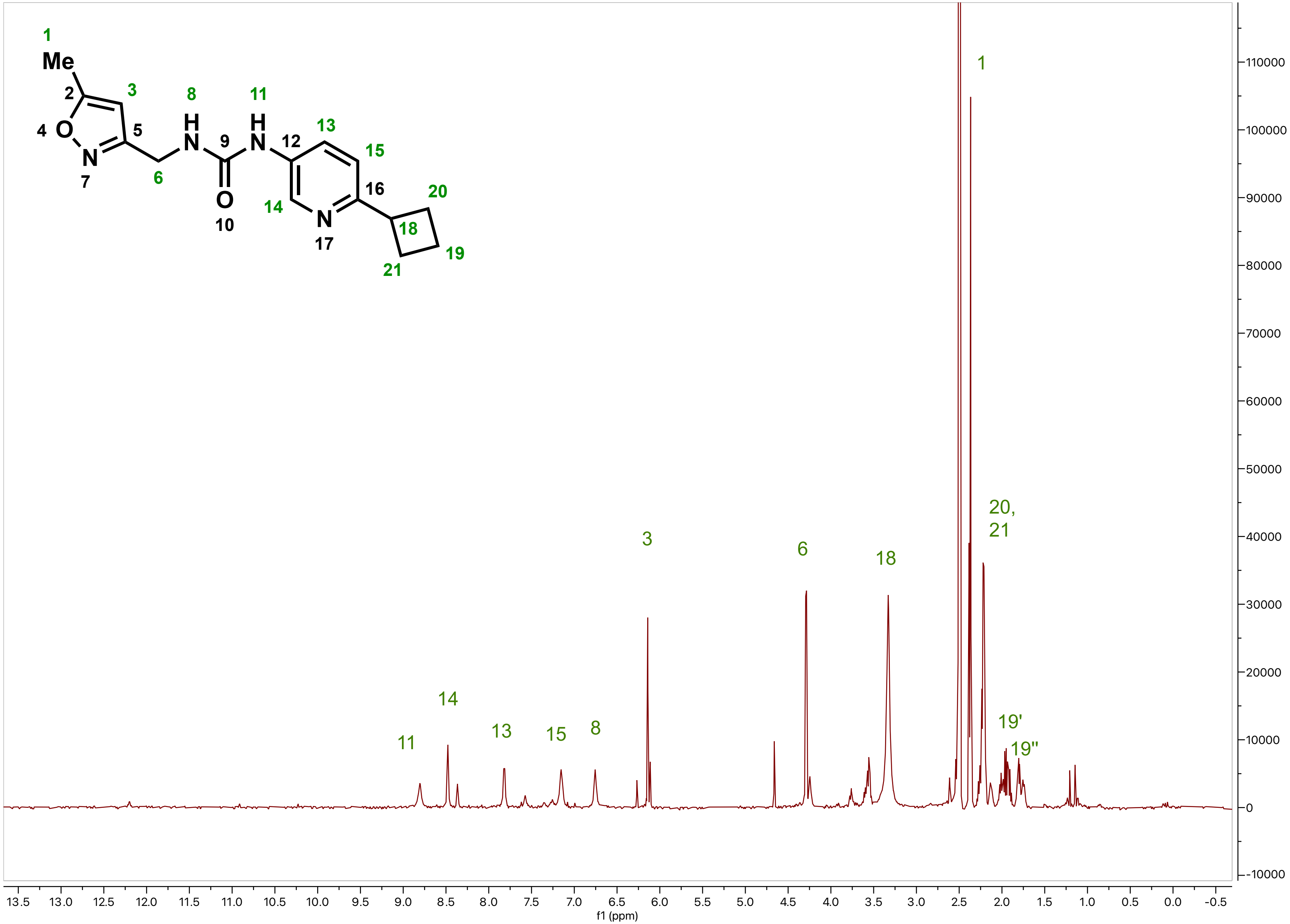

Figure S16: 600 MHz ^{1}H NMR spectrum for compound **S9** in DMSO-d_6. Green numbered atoms are atoms with a potential ^{1}H NMR signal. Green numbered peaks are mapped to their corresponding atom number.

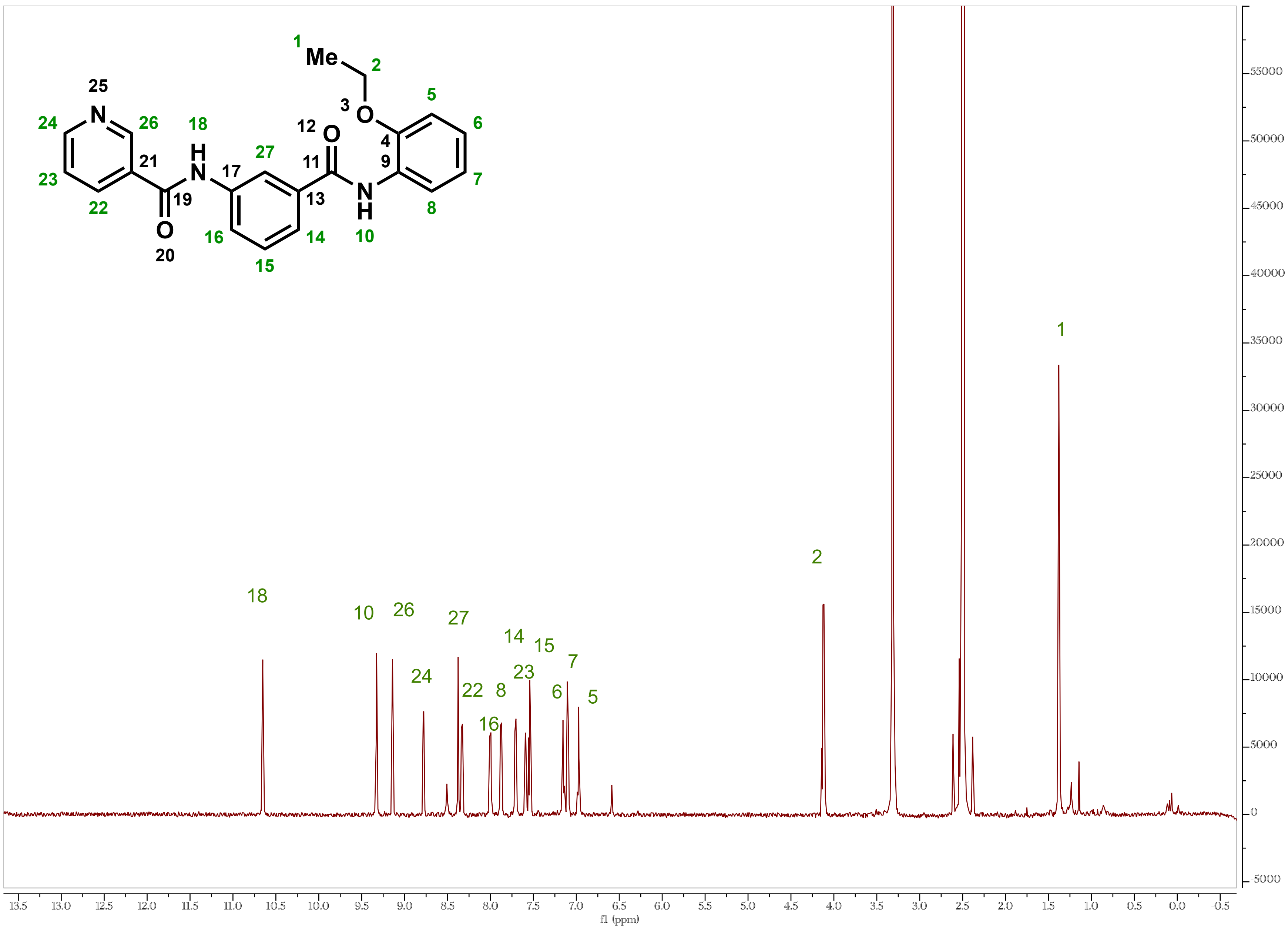

Figure S17: 600 MHz ^{1}H NMR spectrum for compound **7** in DMSO-d_6. Green numbered atoms are atoms with a potential ^{1}H NMR signal. Green numbered peaks are mapped to their corresponding atom number.

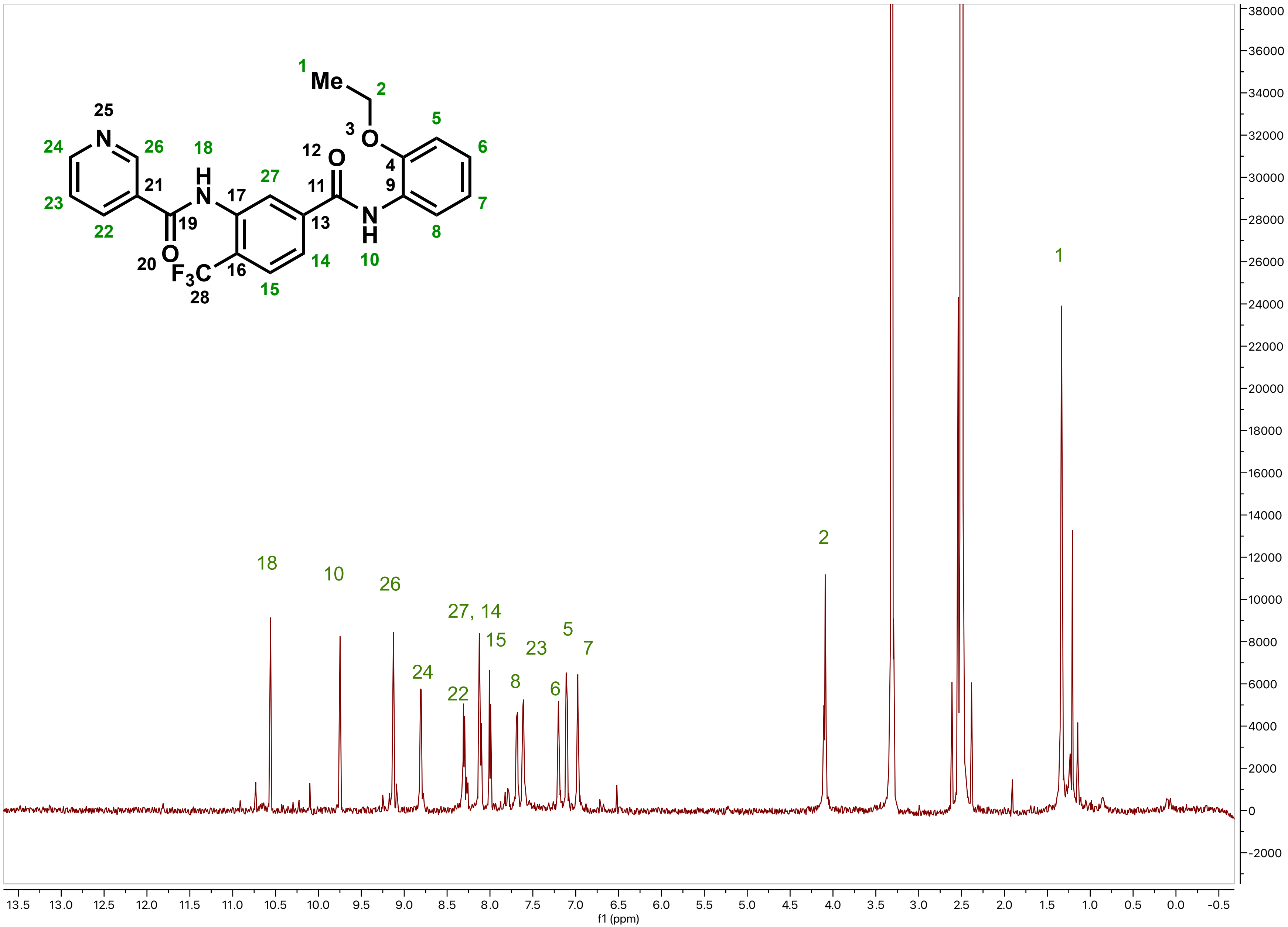

Figure S18: 600 MHz ^{1}H NMR spectrum for compound **S10** in DMSO-d_6. Green numbered atoms are atoms with a potential ^{1}H NMR signal. Green numbered peaks are mapped to their corresponding atom number.

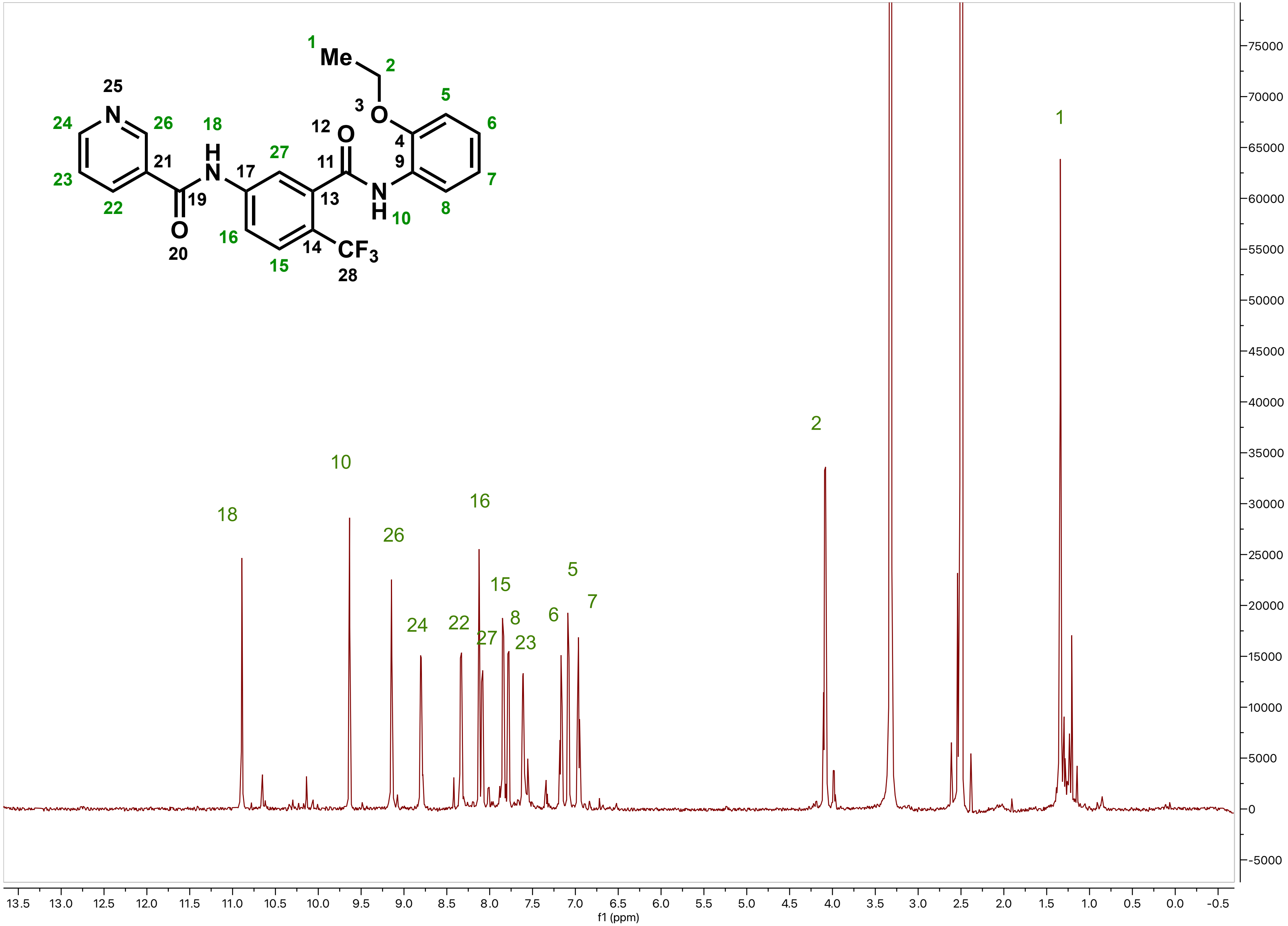

Figure S19: 600 MHz ^{1}H NMR spectrum for compound **S11** in DMSO-d_6. Green numbered atoms are atoms with a potential ^{1}H NMR signal. Green numbered peaks are mapped to their corresponding atom number.

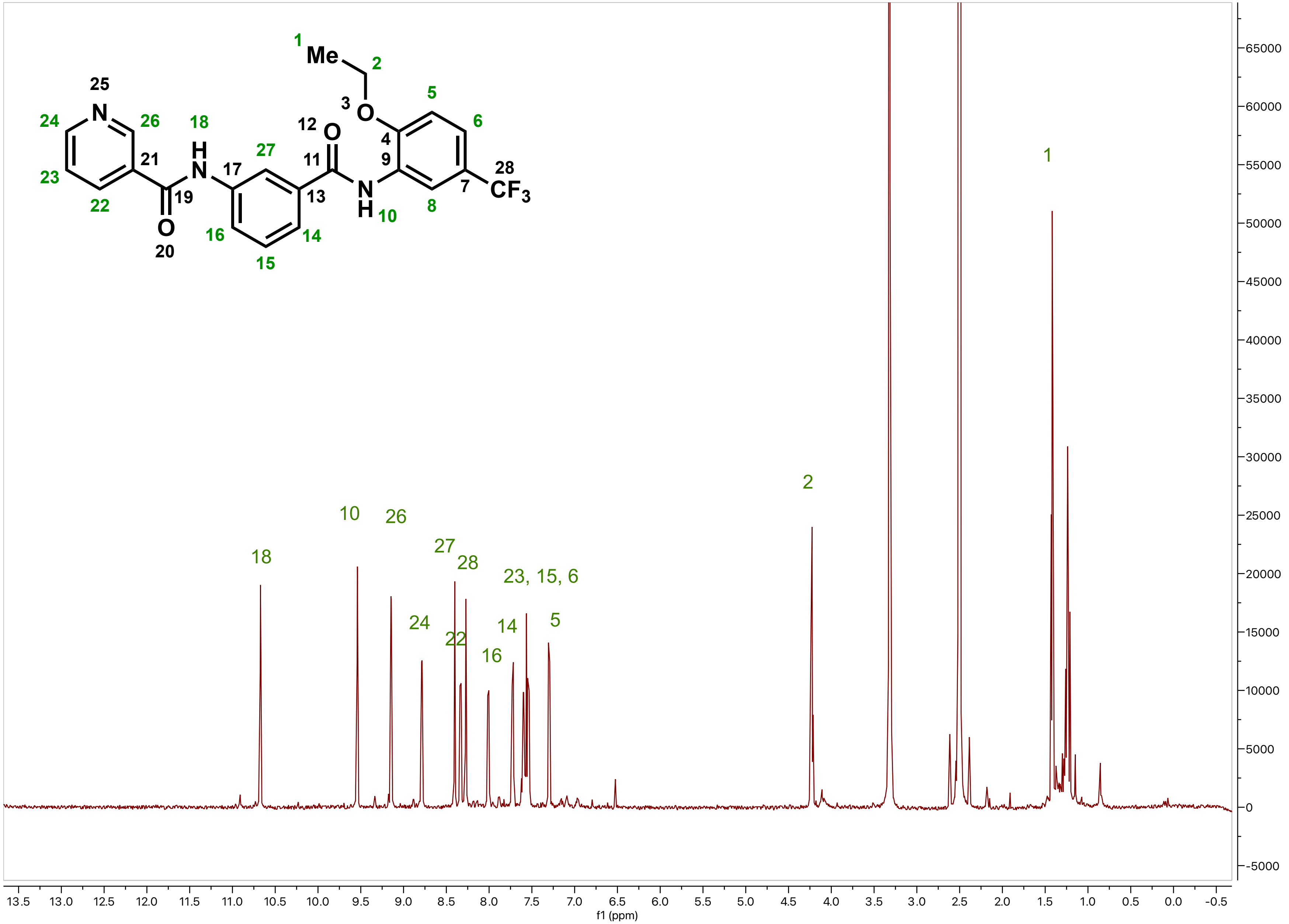

Figure S20: 600 MHz ^{1}H NMR spectrum for compound **S12** in DMSO-d$_6$. Green numbered atoms are atoms with a potential ^{1}H NMR signal. Green numbered peaks are mapped to their corresponding atom number.

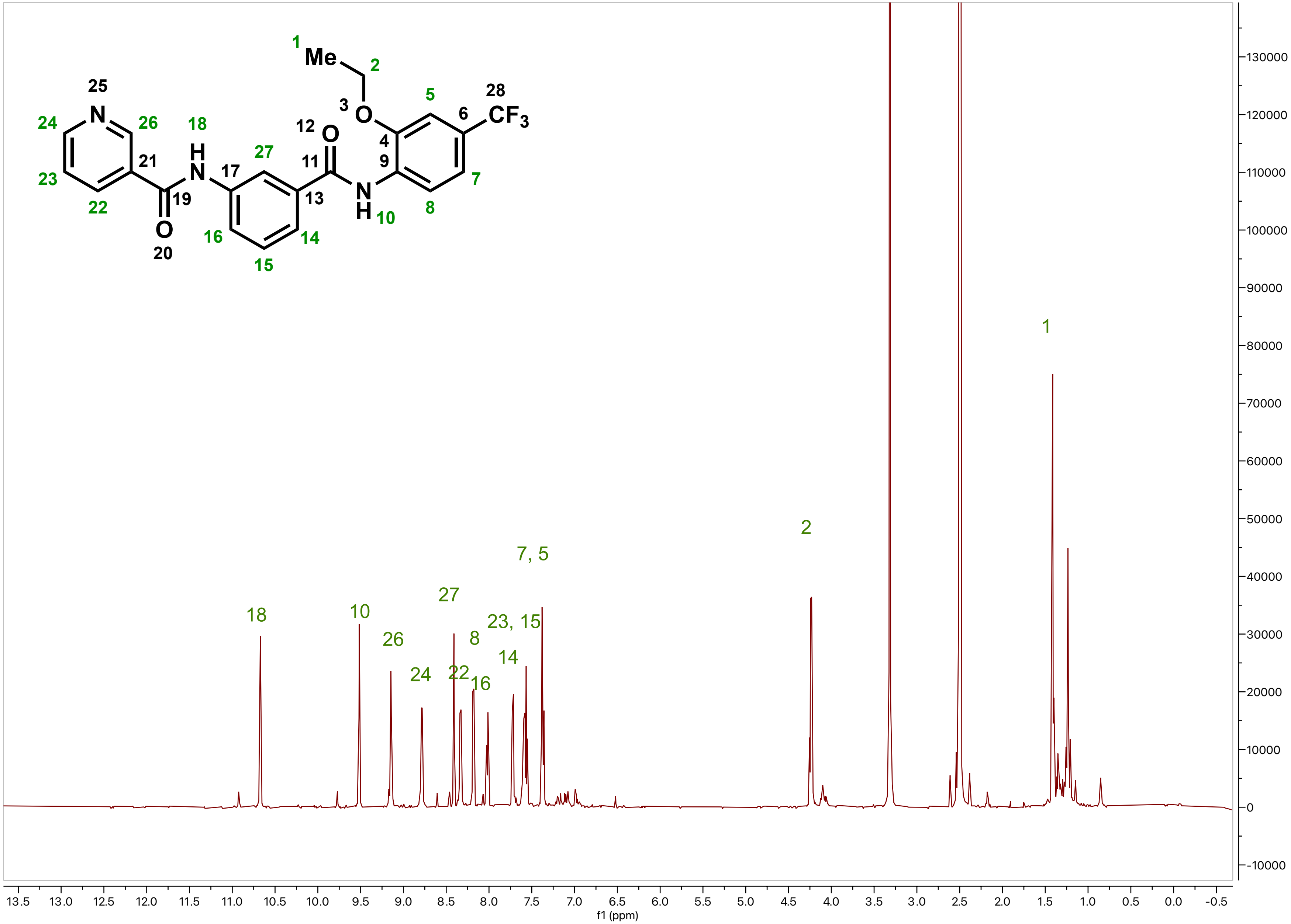

Figure S21: 600 MHz ^{1}H NMR spectrum for compound **S13** in DMSO-d_6. Green numbered atoms are atoms with a potential ^{1}H NMR signal. Green numbered peaks are mapped to their corresponding atom number.

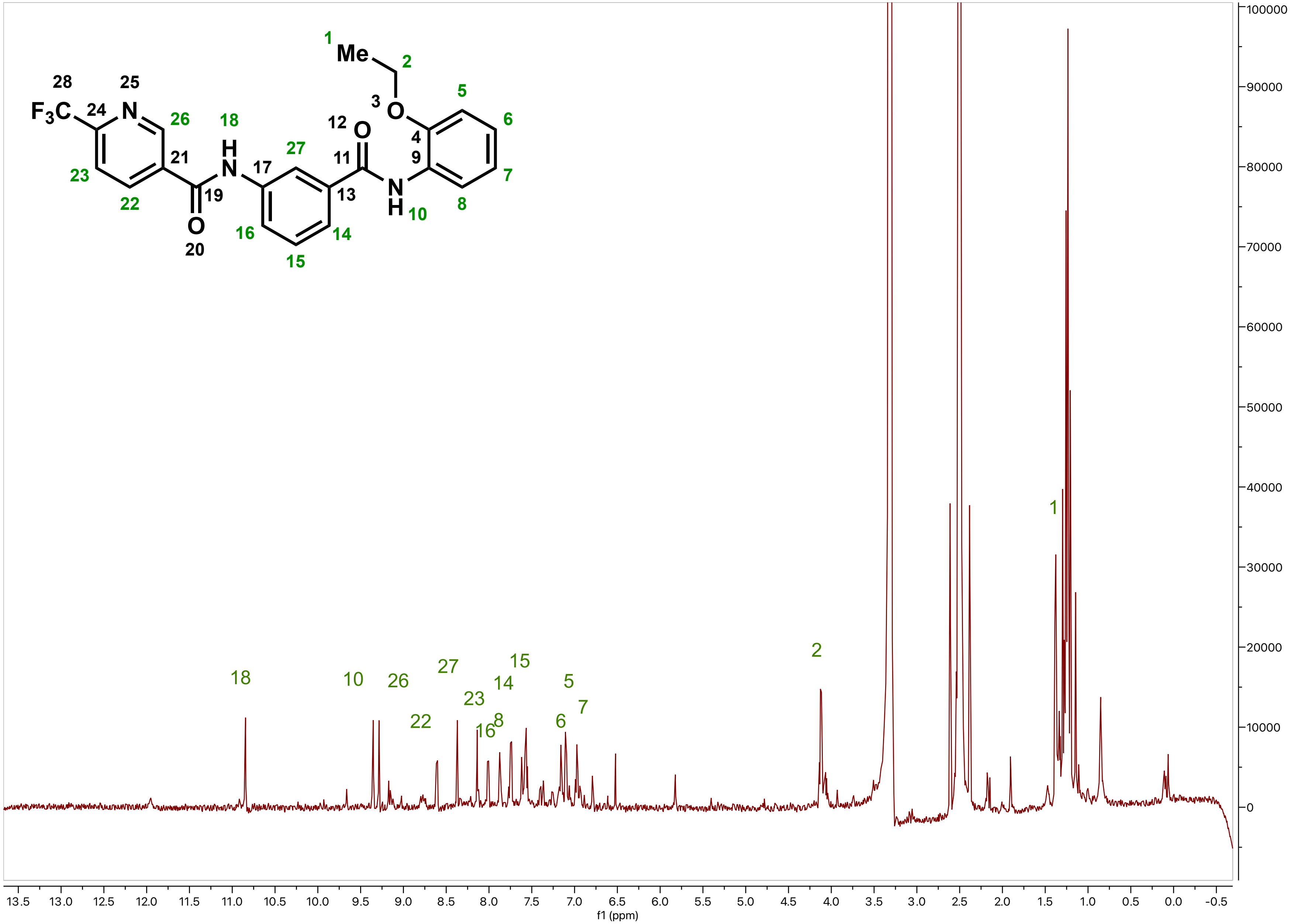

Figure S22: 600 MHz ^{1}H NMR spectrum for compound **S14** in DMSO-d$_6$. Green numbered atoms are atoms with a potential ^{1}H NMR signal. Green numbered peaks are mapped to their corresponding atom number.

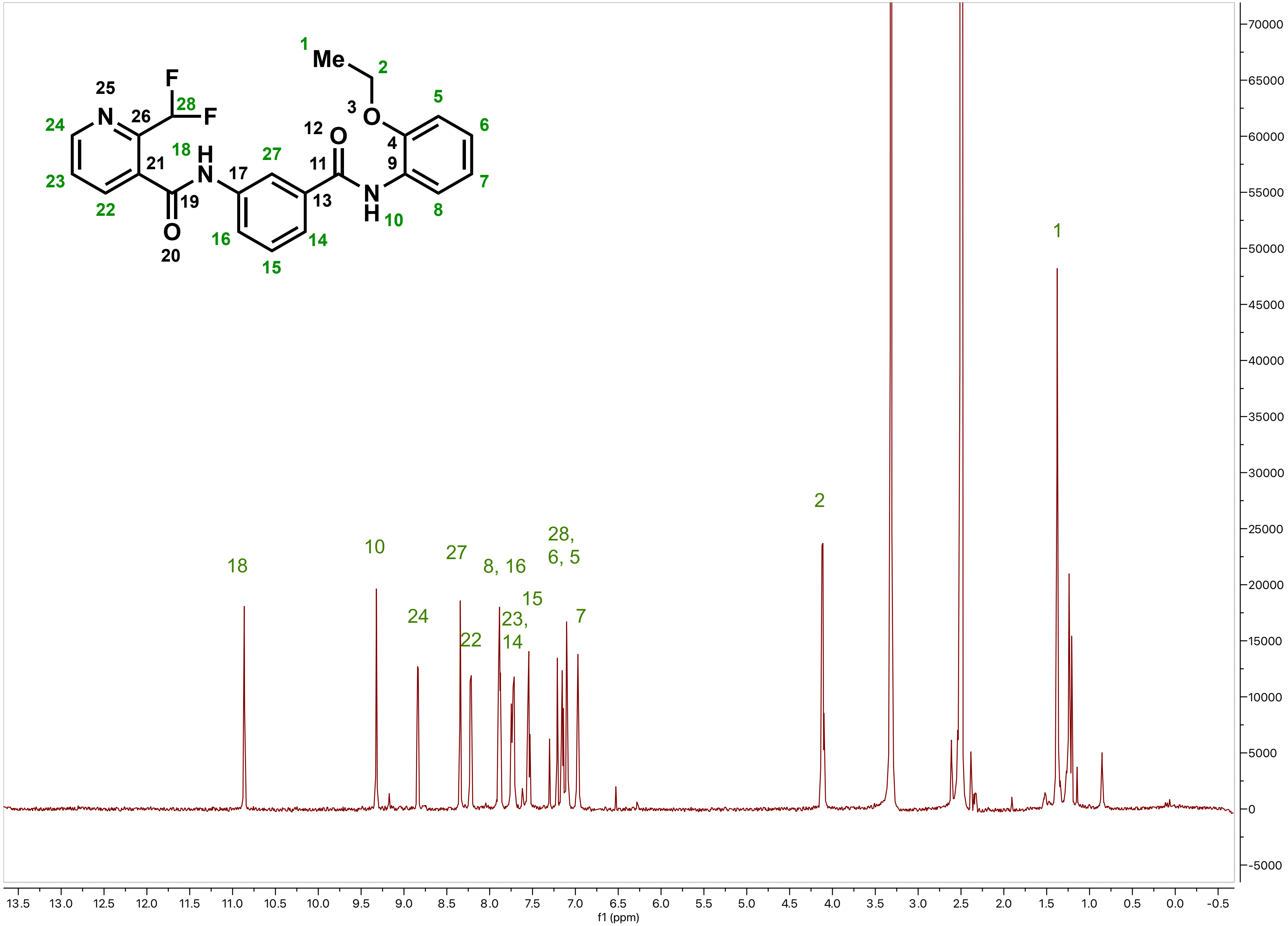

Figure S23: 600 MHz ^{1}H NMR spectrum for compound **S15** in DMSO-d_6. Green numbered atoms are atoms with a potential ^{1}H NMR signal. Green numbered peaks are mapped to their corresponding atom number.

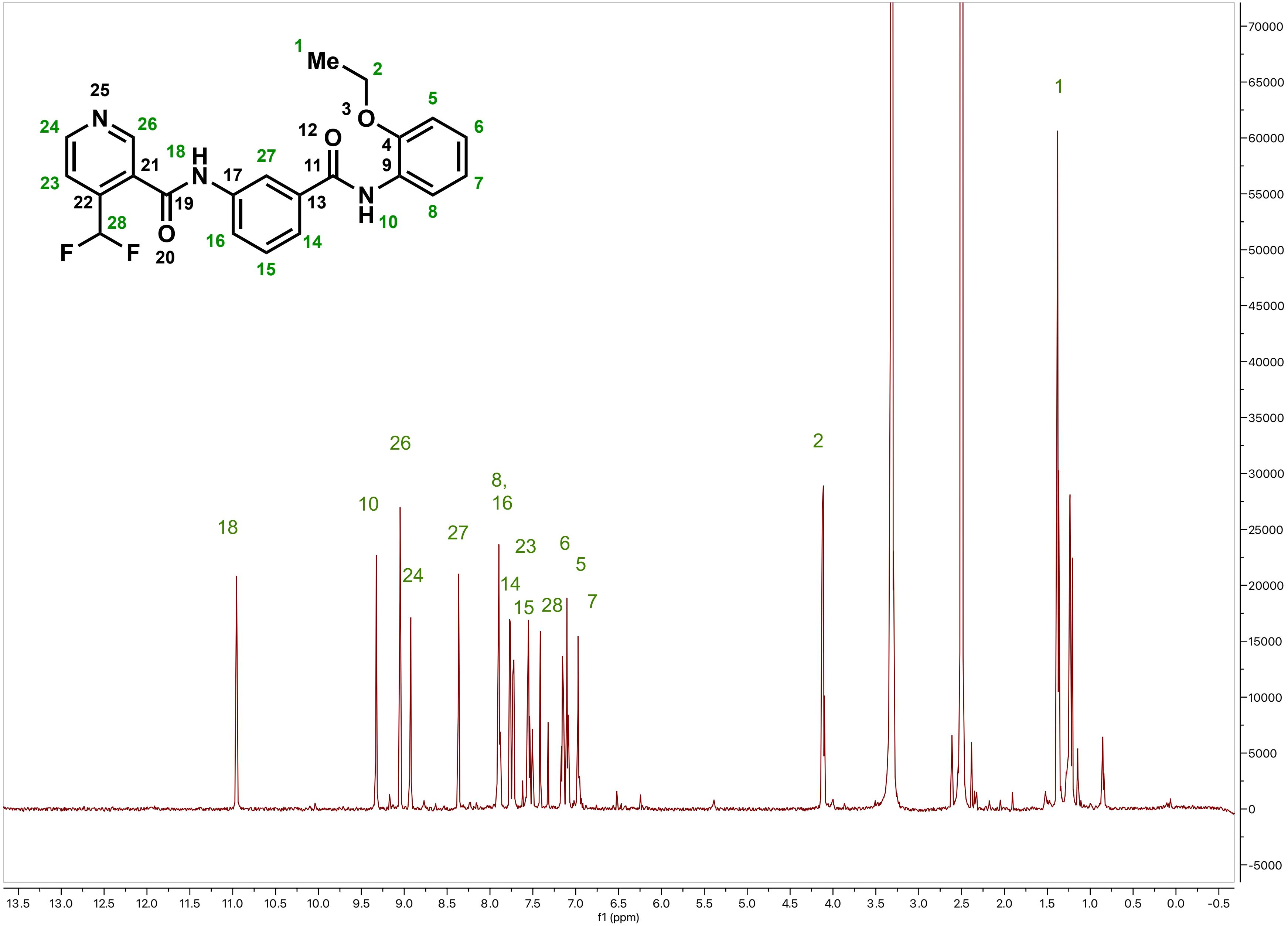

Figure S24: 600 MHz ^{1}H NMR spectrum for compound **S16** in DMSO-d_6. Green numbered atoms are atoms with a potential ^{1}H NMR signal. Green numbered peaks are mapped to their corresponding atom number.

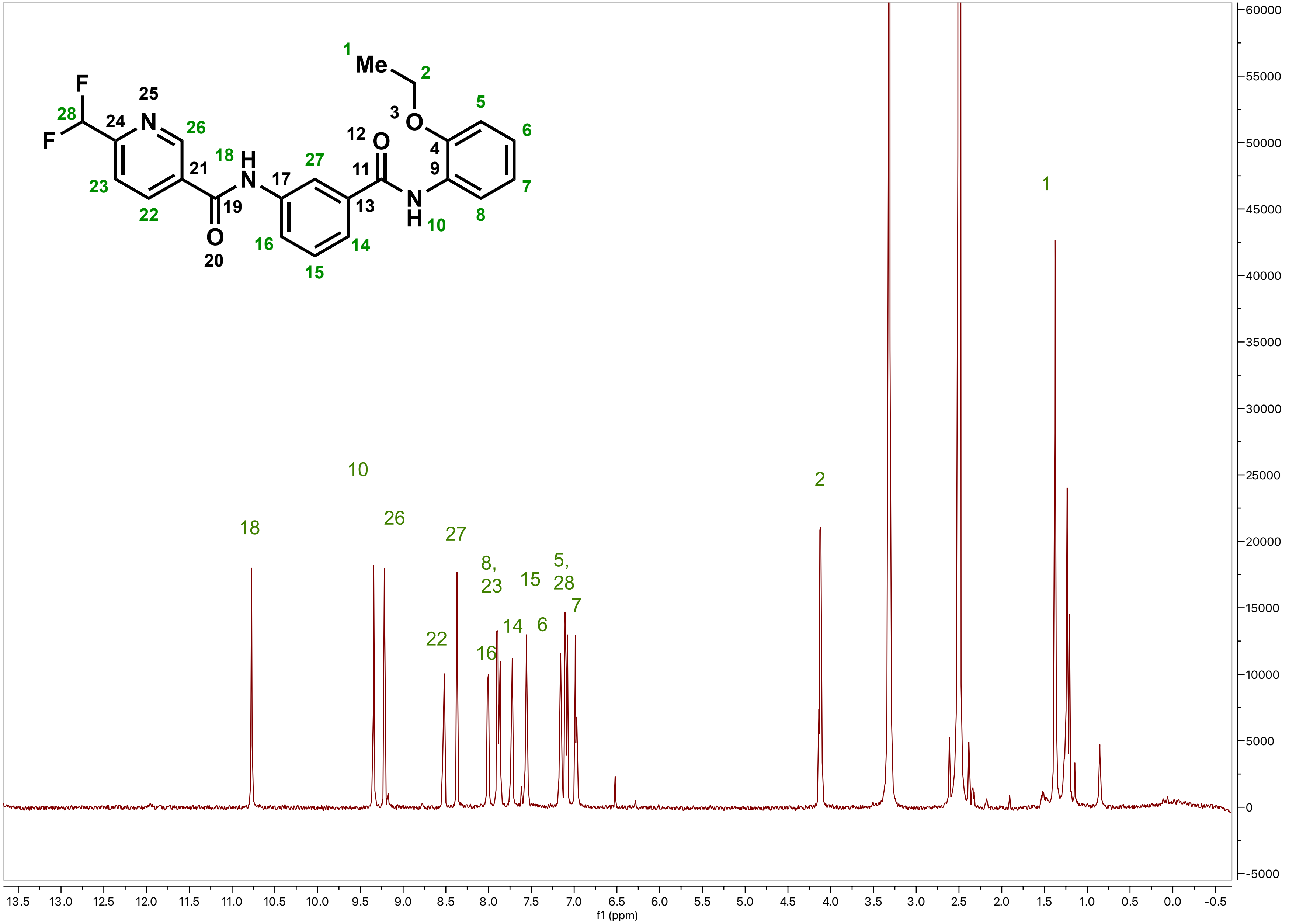

Figure S25: 600 MHz ^{1}H NMR spectrum for compound **S17** in DMSO-d_6. Green numbered atoms are atoms with a potential ^{1}H NMR signal. Green numbered peaks are mapped to their corresponding atom number.

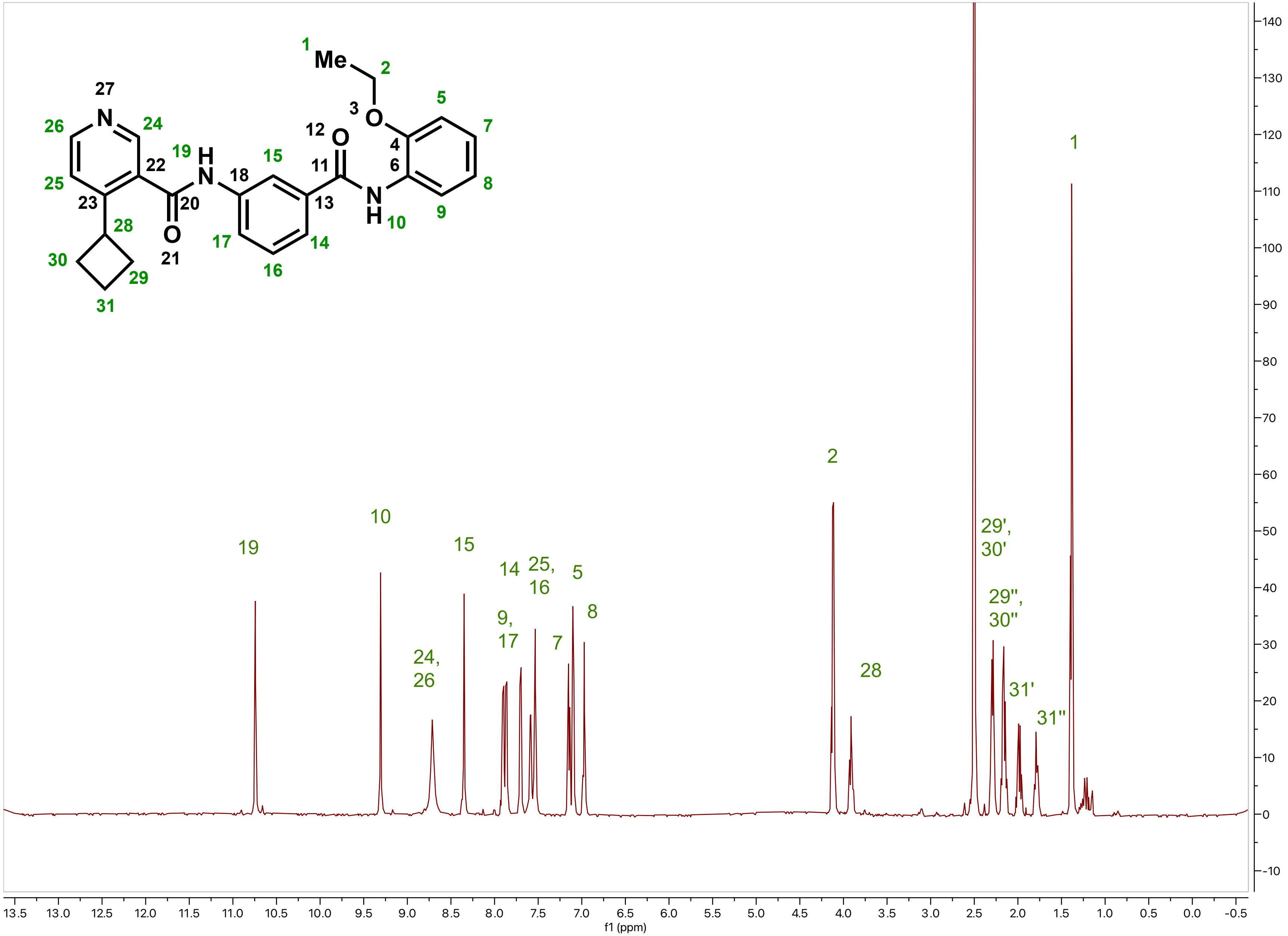

Figure S26: 600 MHz ^{1}H NMR spectrum for compound **S18** in DMSO-d_6. Green numbered atoms are atoms with a potential ^{1}H NMR signal. Green numbered peaks are mapped to their corresponding atom number.

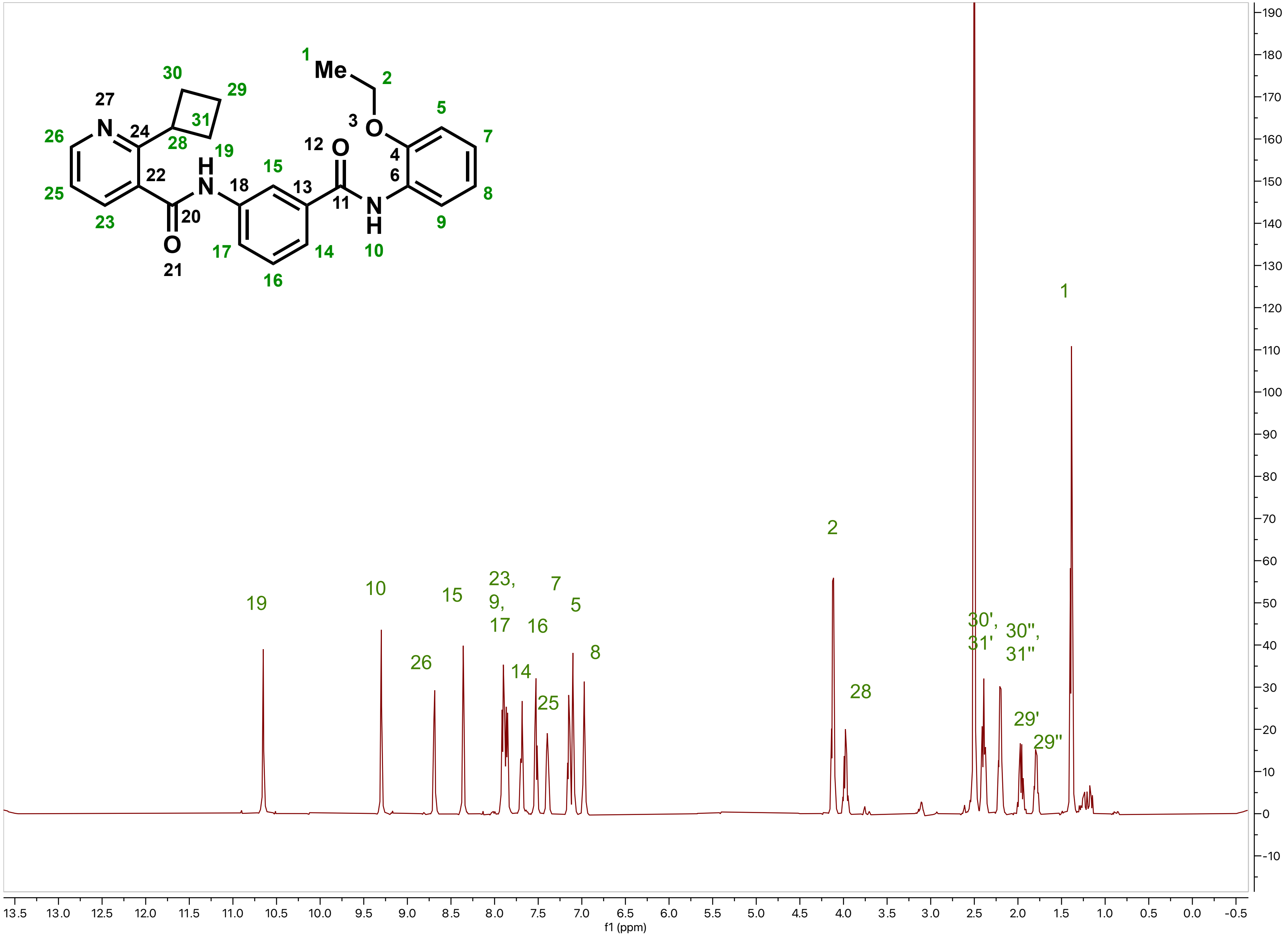

Figure S27: 600 MHz ^{1}H NMR spectrum for compound **S19** in DMSO-d_6. Green numbered atoms are atoms with a potential ^{1}H NMR signal. Green numbered peaks are mapped to their corresponding atom number.

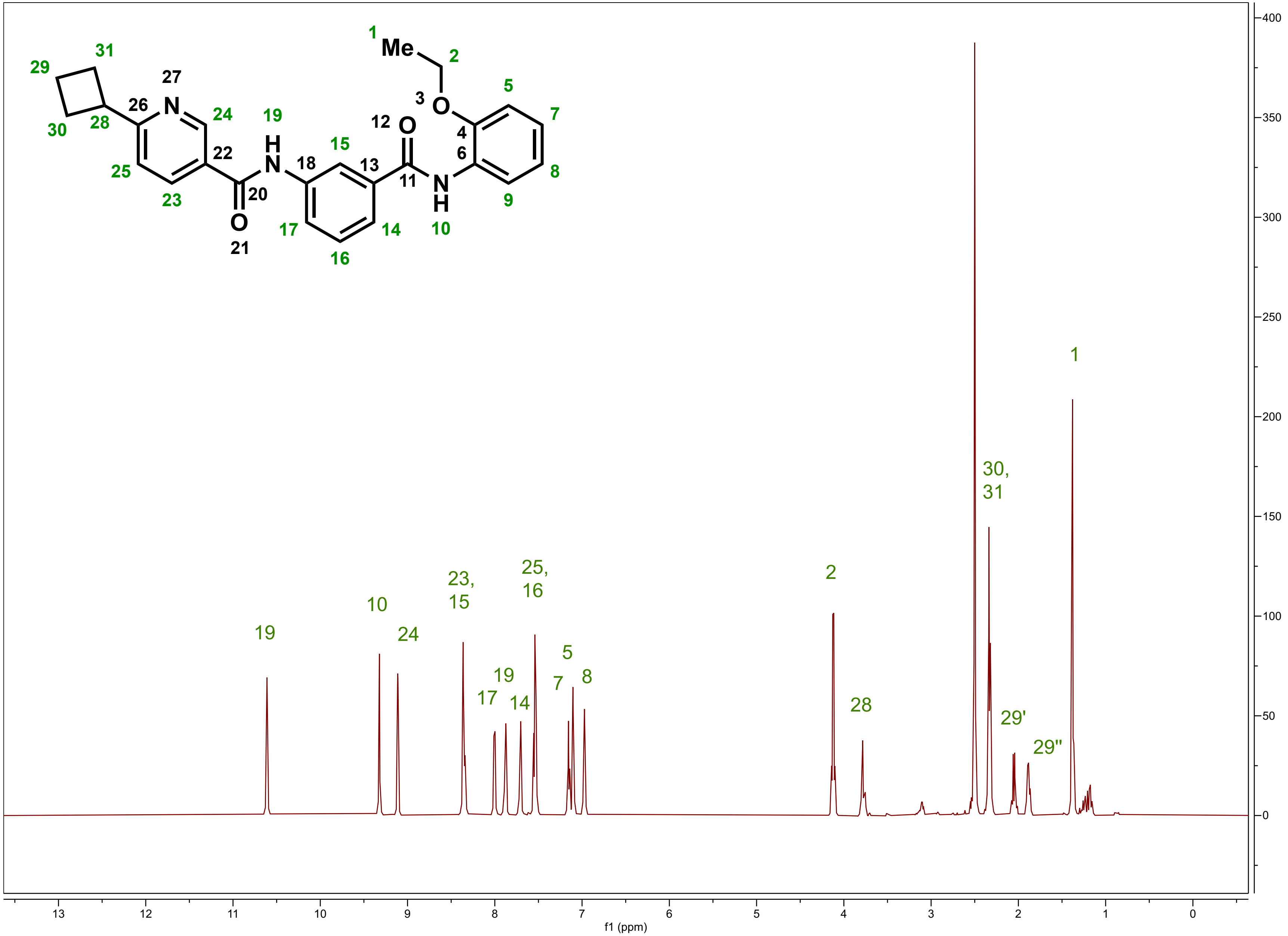

Figure S28: 600 MHz ^{1}H NMR spectrum for compound **S20** in DMSO-d$_6$. Green numbered atoms are atoms with a potential ^{1}H NMR signal. Green numbered peaks are mapped to their corresponding atom number.

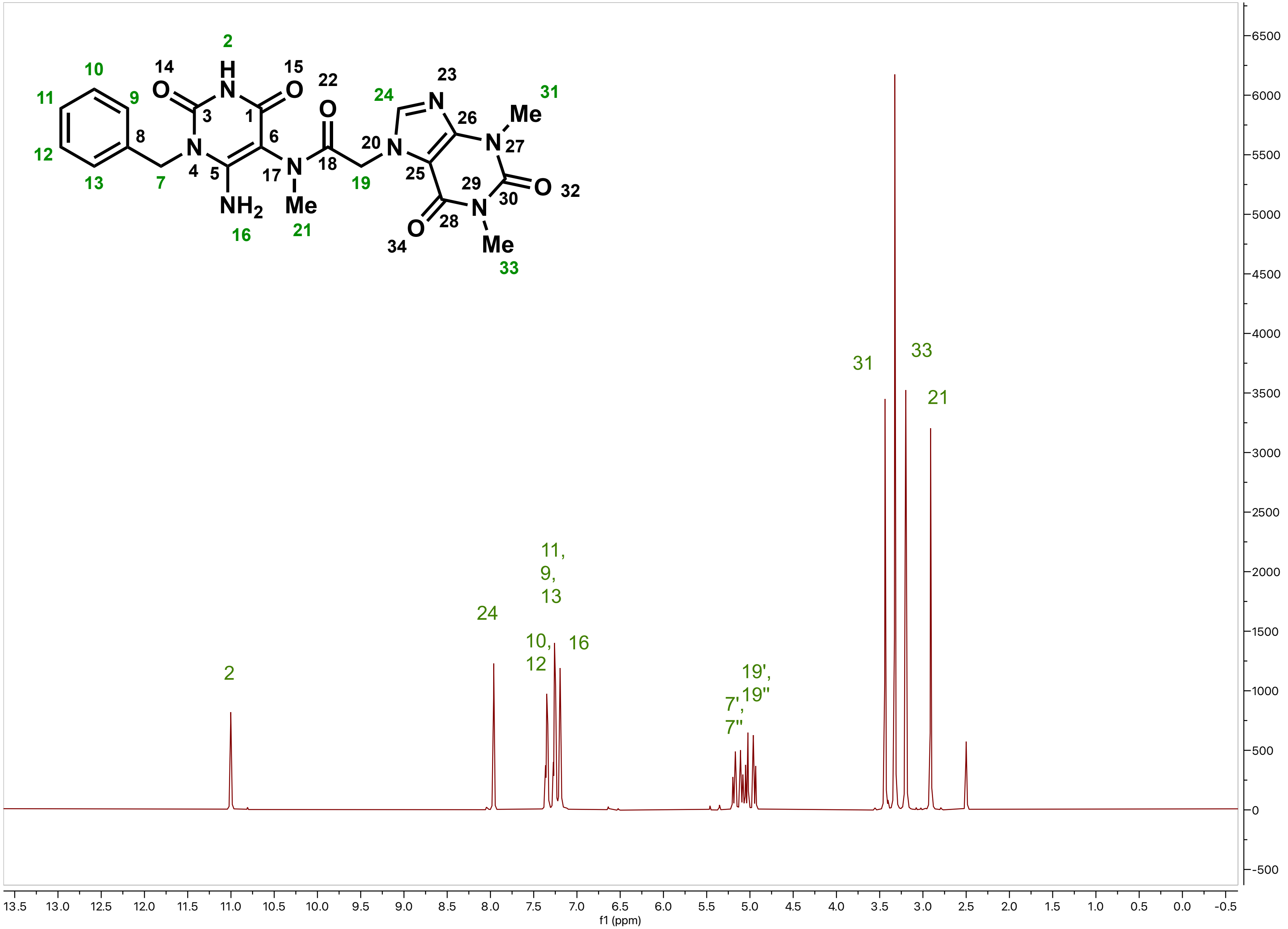

Figure S29: 600 MHz ^{1}H NMR spectrum for compound **8** in DMSO-d$_6$. Green numbered atoms are atoms with a potential ^{1}H NMR signal. Green numbered peaks are mapped to their corresponding atom number.

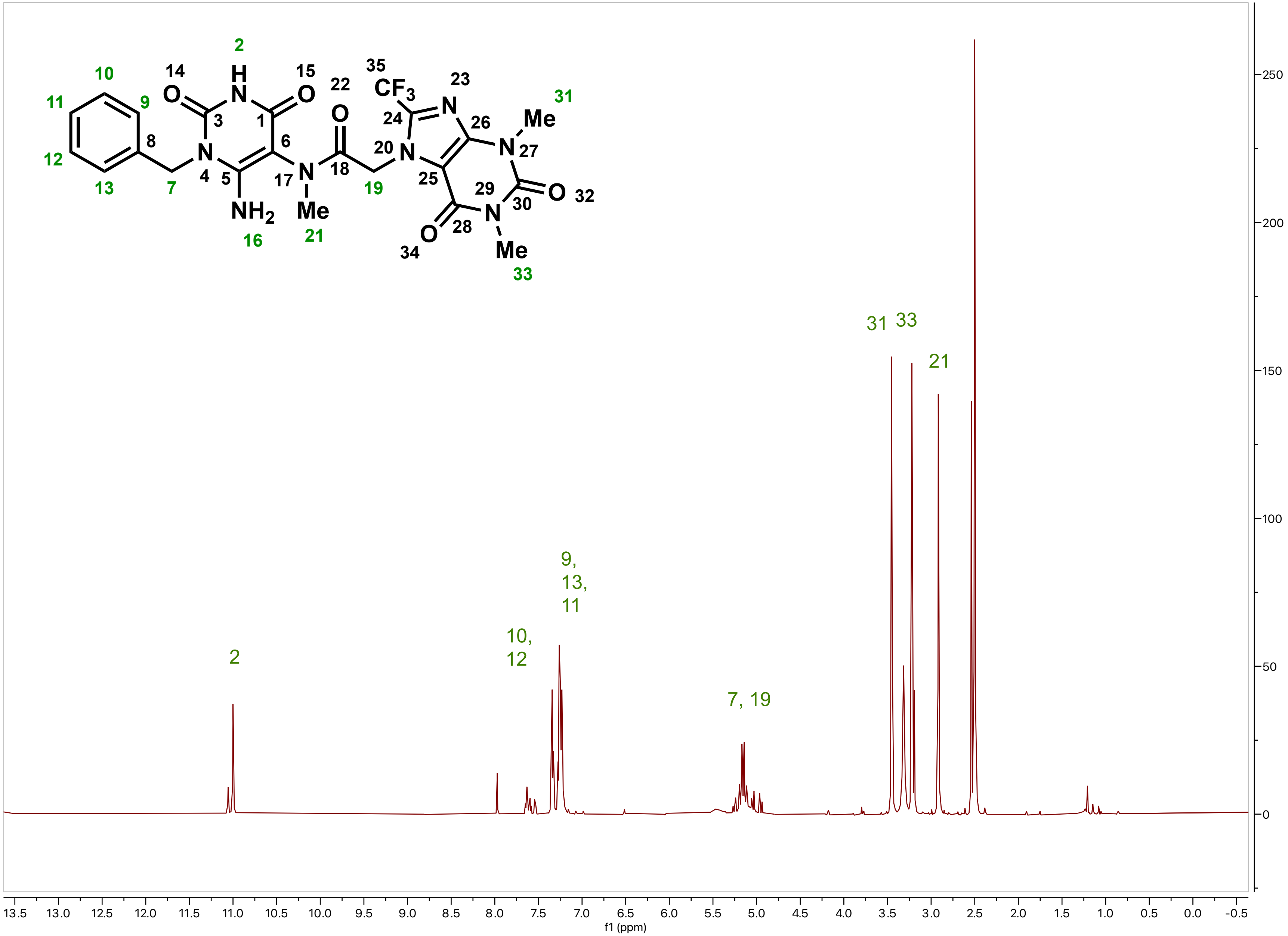

Figure S30: 600 MHz ^{1}H NMR spectrum for compound **S21** in DMSO-d_6. Green numbered atoms are atoms with a potential ^{1}H NMR signal. Green numbered peaks are mapped to their corresponding atom number.

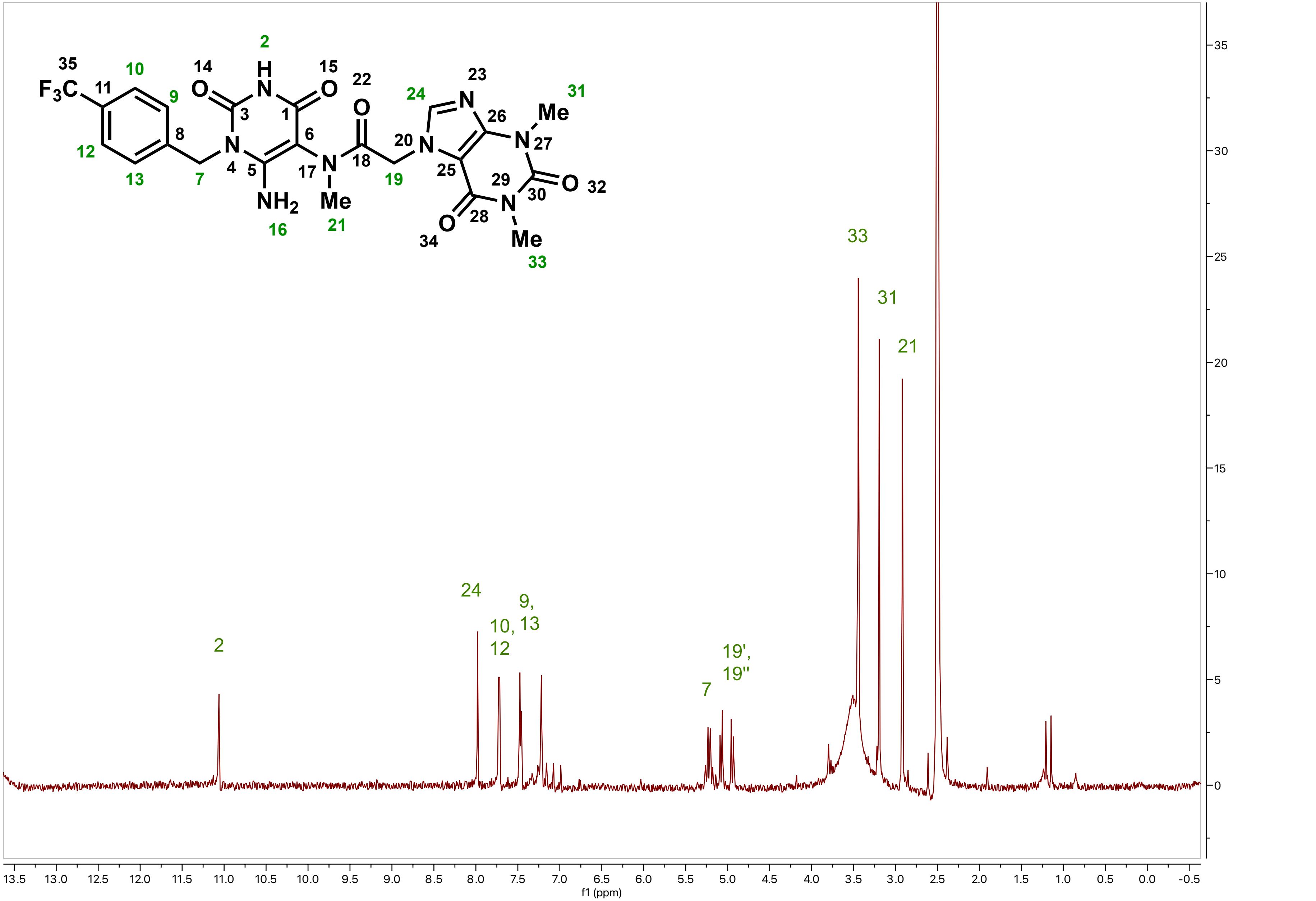

Figure S31: 600 MHz ^{1}H NMR spectrum for compound **S22** in DMSO-d$_6$. Green numbered atoms are atoms with a potential ^{1}H NMR signal. Green numbered peaks are mapped to their corresponding atom number.

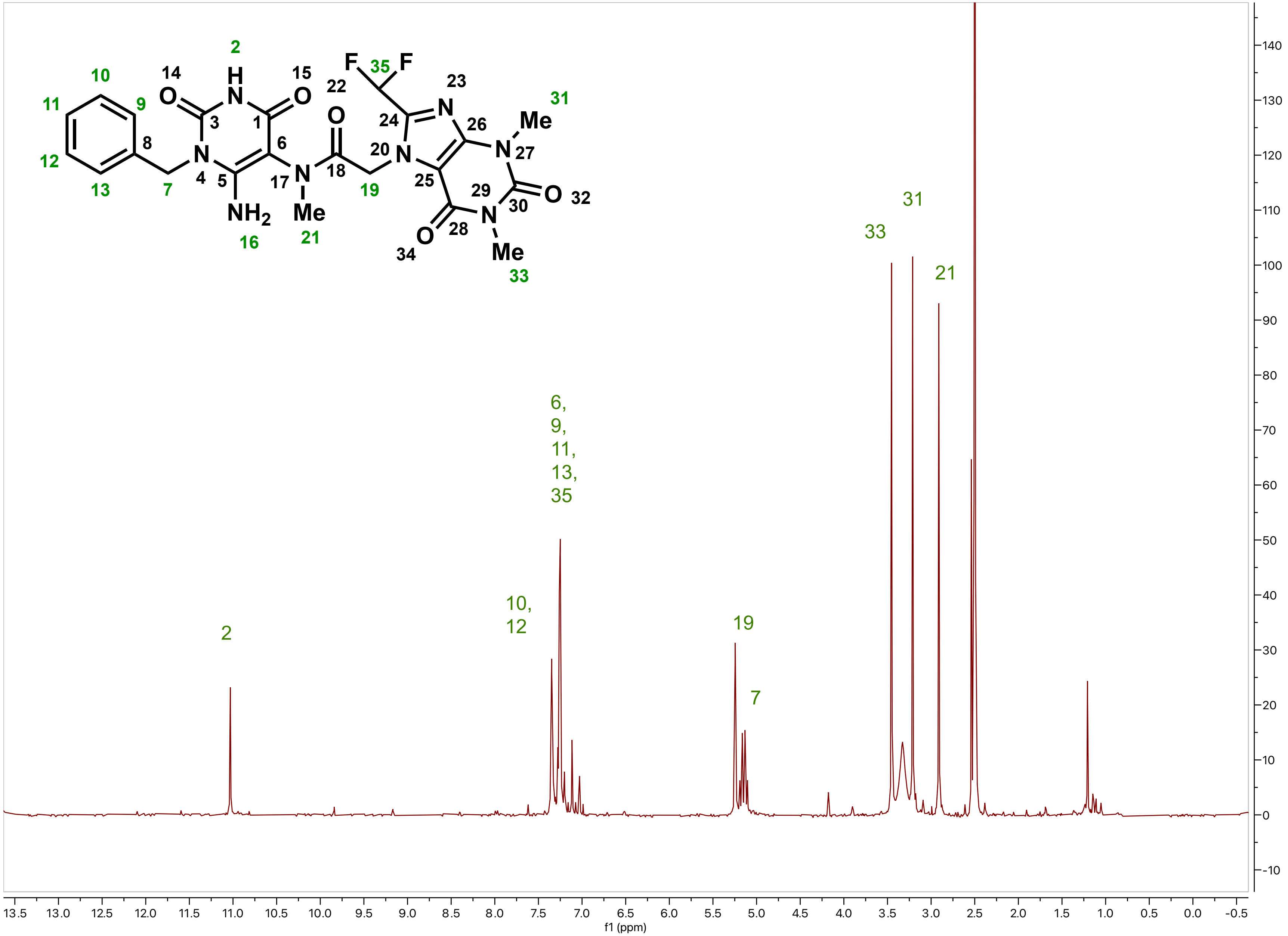

Figure S32: 600 MHz ^{1}H NMR spectrum for compound **S23** in DMSO-d_6. Green numbered atoms are atoms with a potential ^{1}H NMR signal. Green numbered peaks are mapped to their corresponding atom number.

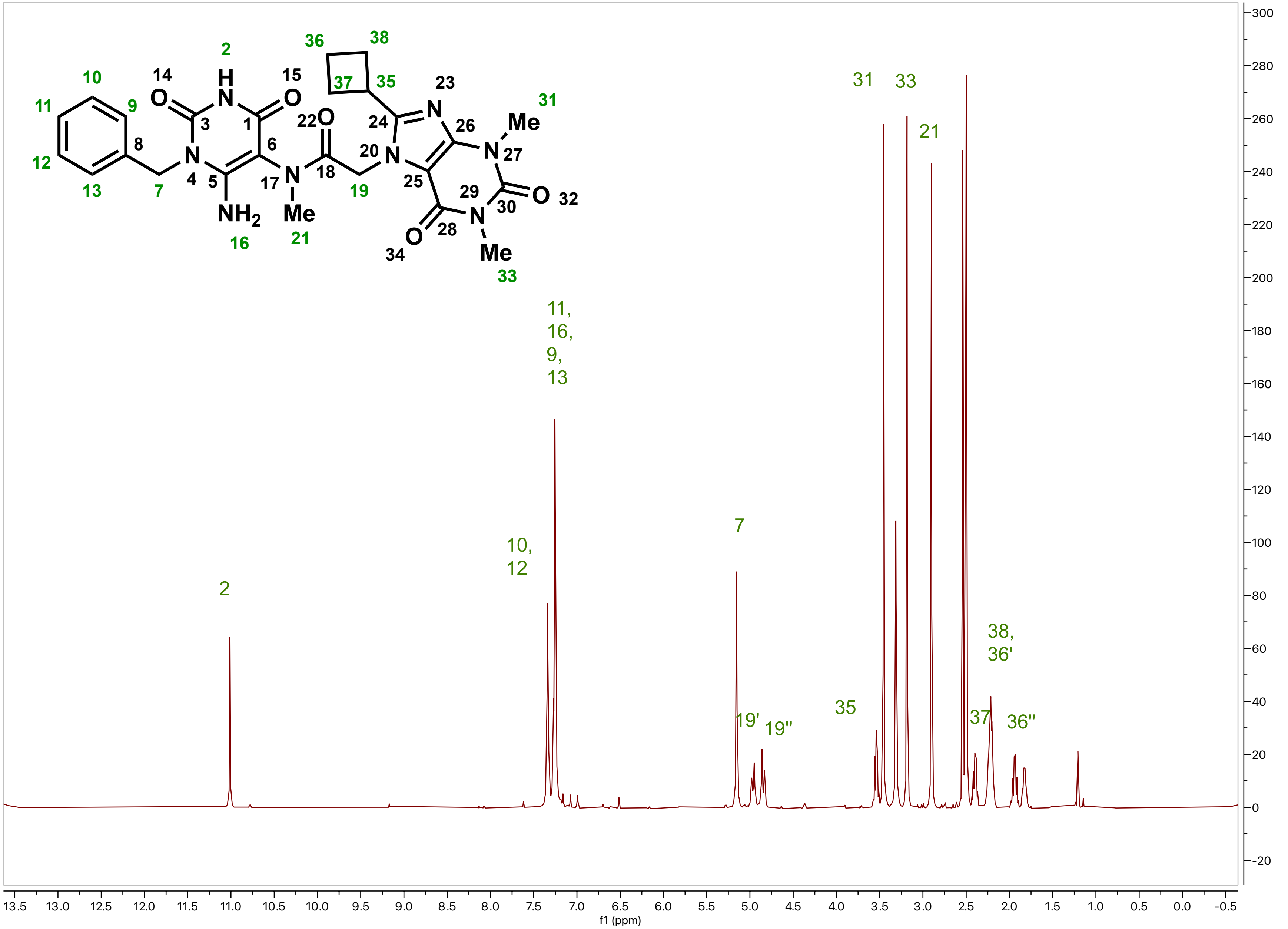

Figure S33: 600 MHz ^{1}H NMR spectrum for compound **S24** in DMSO-d$_6$. Green numbered atoms are atoms with a potential ^{1}H NMR signal. Green numbered peaks are mapped to their corresponding atom number.

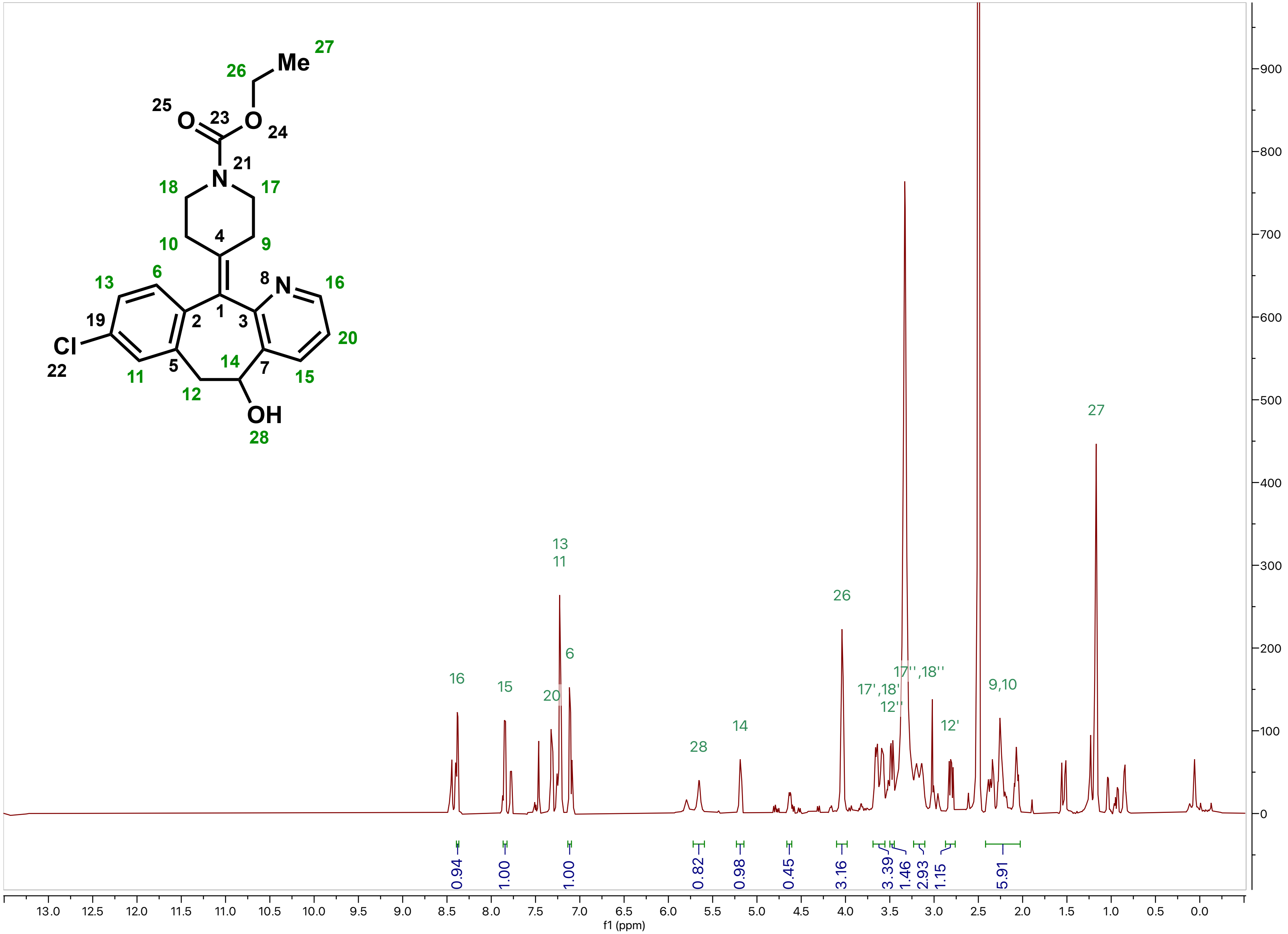

Figure S34: 600 MHz ^{1}H NMR spectrum for compound **S25** in DMSO-d$_6$. Green numbered atoms are atoms with a potential ^{1}H NMR signal. Green numbered peaks are mapped to their corresponding atom number.

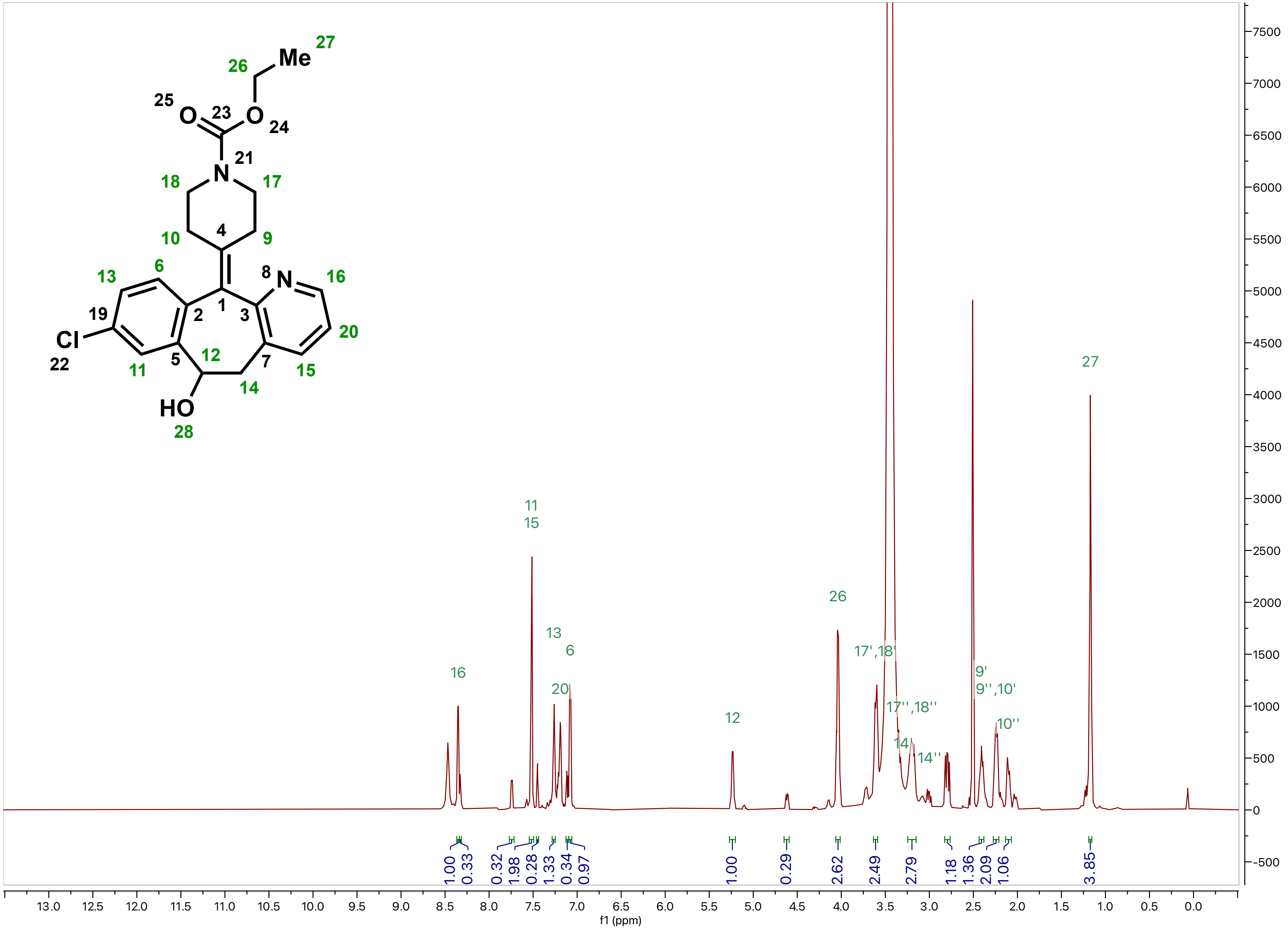

Figure S35: 600 MHz ¹H NMR spectrum for compound **S26** in DMSO-d$_6$. Green numbered atoms are atoms with a potential ¹H NMR signal. Green numbered peaks are mapped to their corresponding atom number.

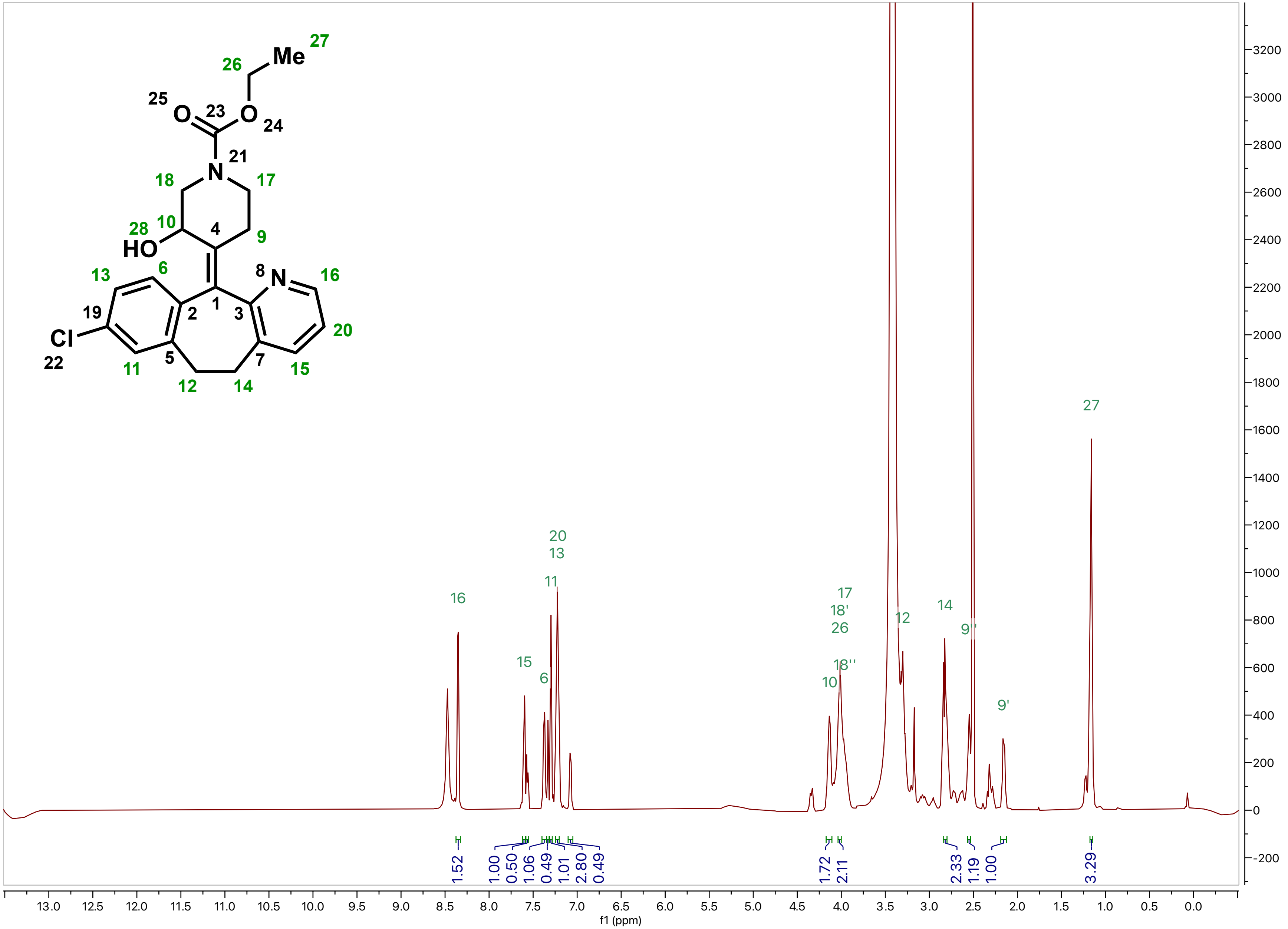

Figure S36: 600 MHz ^{1}H NMR spectrum for compound **S27** in DMSO-d_6. Green numbered atoms are atoms with a potential ^{1}H NMR signal. Green numbered peaks are mapped to their corresponding atom number.

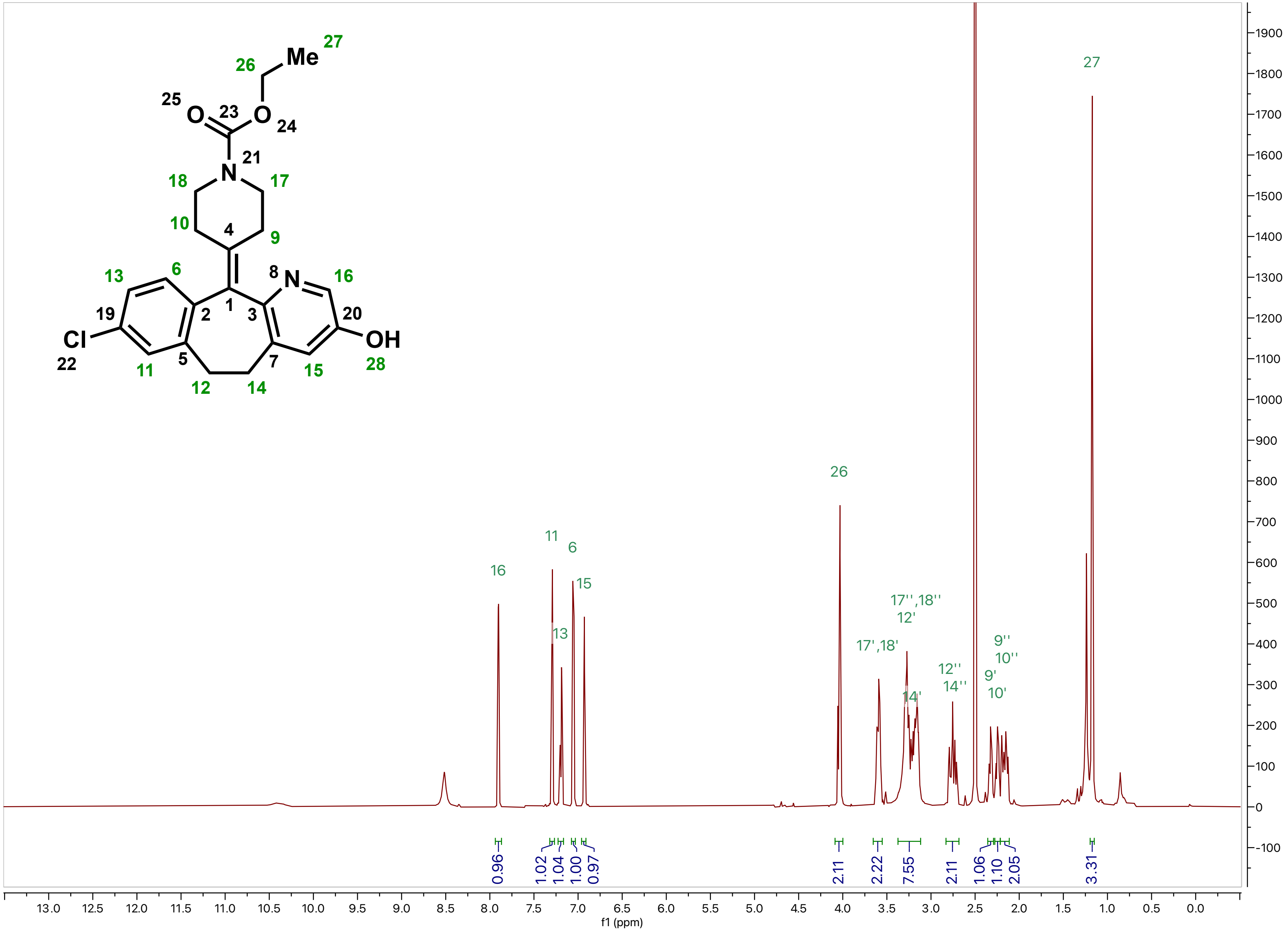

Figure S37: 600 MHz ^{1}H NMR spectrum for compound **S28** in DMSO-d_6. Green numbered atoms are atoms with a potential ^{1}H NMR signal. Green numbered peaks are mapped to their corresponding atom number.

Supplementary References:

1 Lall, M. S. *et al.* Late-Stage Lead Diversification Coupled with Quantitative Nuclear Magnetic Resonance Spectroscopy to Identify New Structure–Activity Relationship Vectors at Nanomole-Scale Synthesis: Application to Loratadine, a Human Histamine H1 Receptor Inverse Agonist. *Journal of Medicinal Chemistry* **63**, 7268-7292, doi:10.1021/acs.jmedchem.0c00483 (2020).

2 http://openmopac.net/.

3 Hjorth Larsen, A. *et al.* The atomic simulation environment—a Python library for working with atoms. *Journal of Physics: Condensed Matter* **29**, 273002, doi:10.1088/1361-648X/aa680e (2017).

4 Batatia, I., Kovács, D. P., Simm, G. N., Ortner, C. & Csányi, G. Mace: Higher order equivariant message passing neural networks for fast and accurate force fields. *arXiv preprint arXiv:2206.07697* (2022).

5 Seritan, S. *et al.* TeraChem: A graphical processing unit-accelerated electronic structure package for large-scale ab initio molecular dynamics. *WIREs Computational Molecular Science* **11**, e1494, doi:https://doi.org/10.1002/wcms.1494 (2021).
